# NLRC5 overexpression in ovarian tumors remodels the tumor microenvironment and increases T-cell reactivity toward autologous tumor-associated antigens

**DOI:** 10.3389/fimmu.2023.1295208

**Published:** 2024-01-03

**Authors:** Galaxia M. Rodriguez, Edward Yakubovich, Humaira Murshed, Vincent Maranda, Kristianne J.C. Galpin, Alison Cudmore, Andrew M. R. Hanna, Elizabeth Macdonald, Shashankan Ramesh, Kenneth Garson, Barbara C. Vanderhyden

**Affiliations:** ^1^ Cancer Therapeutics Program, Ottawa Hospital Research Institute, Ottawa, ON, Canada; ^2^ Department of Cellular and Molecular Medicine, University of Ottawa, Ottawa, ON, Canada

**Keywords:** ovarian cancer, tumor immunogenicity, MHC I, NLRC5, tumor microenvironment, infected cell vaccine

## Abstract

**Introduction:**

Epithelial ovarian cancer (OC) stands as one of the deadliest gynecologic malignancies, urgently necessitating novel therapeutic strategies. Approximately 60% of ovarian tumors exhibit reduced expression of major histocompatibility complex class I (MHC I), intensifying immune evasion mechanisms and rendering immunotherapies ineffective. NOD-like receptor CARD domain containing 5 (NLRC5) transcriptionally regulates MHC I genes and many antigen presentation machinery components. We therefore explored the therapeutic potential of NLRC5 in OC.

**Methods:**

We generated OC cells overexpressing NLRC5 to rescue MHC I expression and antigen presentation and then assessed their capability to respond to PD-L1 blockade and an infected cell vaccine.

**Results:**

Analysis of microarray datasets revealed a correlation between elevated NLRC5 expression and extended survival in OC patients; however, NLRC5 was scarcely detected in the OC tumor microenvironment. OC cells overexpressing NLRC5 exhibited slower tumor growth and resulted in higher recruitment of leukocytes in the TME with lower CD4/CD8 T-cell ratios and increased activation of T cells. Immune cells from peripheral blood, spleen, and ascites from these mice displayed heightened activation and interferon-gamma production when exposed to autologous tumor-associated antigens. Finally, as a proof of concept, NLRC5 overexpression within an infected cell vaccine platform enhanced responses and prolonged survival in comparison with control groups when challenged with parental tumors.

**Discussion:**

These findings provide a compelling rationale for utilizing NLRC5 overexpression in “cold” tumor models to enhance tumor susceptibility to T-cell recognition and elimination by boosting the presentation of endogenous tumor antigens. This approach holds promise for improving antitumoral immune responses in OC.

## 1 Introduction

Tumor recognition by T cells is essential for the development of effective antitumoral responses. Cytotoxic T lymphocytes (CTLs), main players of antitumoral immunity, fundamentally need two factors to mediate their effector antitumoral functions: i) a proper inflammatory environment and ii) presentation of tumor-associated antigens (TAAs) by antigen-presenting cells, such as dendritic (DCs) and B cells which present TAAs loaded on major histocompatibility complex class I molecules (MHC I; HLA for human, H2 complex for mouse). Frequently, cancer cells evade antitumoral immunity by losing immunogenicity, a hallmark of cancer (1), which can be triggered by down-modulation or loss of expression of MHC I and/or the antigen processing and presentation machinery (APM) (2–4).

Epithelial ovarian cancer (OC) is the most lethal gynecologic malignancy. The disease is normally diagnosed after widespread dissemination, and standard treatment combines debulking surgery with platinum-based chemotherapy. Despite initial response to these treatments, most patients undergo relapse with peritoneal carcinomatosis, resulting in a 5-year mortality of >55% (5). Disappointingly, less than 15% of OC patients show any sign of response to immune checkpoint inhibitors (ICIs) (6), with patients with preexisting antitumoral immunity presenting a better response (7). This supports the hypothesis that the dearth of immune cells in the OC tumor microenvironment (TME) limits responsiveness to immunotherapy. Moreover, expression of MHC I genes is impaired in up to 60% of OC patients (8, 9). This loss of expression participates in the development of a “cold” TME, thereby limiting response to immunotherapies such as ICIs. Strategies to restore MHC I expression thus hold promise for improving OC immunogenicity (10) since high CTL infiltration is associated with improved prognosis (11, 12), along with abundance of CD4+ T cells (Th) (13) and B cells (14) potentially supporting CTL responses.

In support of this reasoning, the presence of T cells specific for neo-antigens expressed by OC cells is strongly associated with increased survival (15–17). However, neo-epitope-specific T cells are mainly found in patients with elevated APM signature (17) which is not surprising given the widespread defect in antigen presentation found in ovarian cancer cells. NLRC5 (NLR CARD domain-containing 5 or CITA) is a critical regulator of MHC I genes, inducing the expression of both classical MHC I (i.e., HLA-A, -B, and -C), and non-classical class I (i.e., HLA-E, -F, and -G) molecules but also main components of the APM pathway like β2-microglobulin (B2M), immunoproteasome components (PSMB9, i.e., LMP2), and peptide transporters (TAP1) (18, 19). Recently, we (Rodriguez) provided the first evidence that enhanced NLRC5-driven MHC I expression increases the susceptibility of “hot” melanoma tumors to CD8+ T cell recognition (20). These findings raise the exciting possibility of using NLRC5-induced expression of MHC I to stimulate antitumor immunity, potentially increasing the pool of TAAs presented to preexisting antitumoral CTLs. Therefore, we investigated the therapeutic potential of NLRC5 in OC, a “cold” and poorly immunogenic disease.

Defects in NLRC5 expression or function are observed in many types of human tumors, including OC, with NLRC5 being the most downregulated gene among immune-related genes in cancer (21). Therapeutic strategies striving to achieve HLA I cloning and the targeted delivery of antigens to ovarian cancer cells are faced with various limitations. These limitations primarily revolve around challenges related to the numerous HLA haplotypes, polymorphisms, and the potential loss of expression of the intended antigens (22, 23).

In this study, we sought to investigate the therapeutic potential of NLRC5 overexpression in OC immunogenicity and response to treatment. To this end, we first examined NLRC5 expression and its main target genes using a human OC single-cell RNA-seq (scRNA-seq) database (24). Secondly, we assessed the effect of NLRC5 overexpression *in vitro* and *in vivo* using the syngeneic murine ID8-*Trp53^−/−^* model and found that restoring NLRC5 expression in OC cells rescues MHC I expression and T-cell effector functions, generating a “hot” TME by decreasing local immunosuppression. Furthermore, we found that NLRC5+ ovarian tumors respond better to PD-L1 blockade. Finally, we showed that when used in an infected cell vaccine, NLRC5 increases response to treatment by enhancing the T-cell pool recognizing TAAs.

## 2 Materials and methods

### 2.1 Primary cell culture and cell lines

*Mouse cell lines:* ID8-*Trp53^−/−^* F3 (ID8-p53−/−, or F3) cells were obtained from Dr. Iain McNeish as described elsewhere (25). STOSE cells were generated in our laboratory and were characterized previously (26, 27). ID8-p53−/−GLuc were generated in our laboratory by lentiviral transduction with pMCS-Gaussia Luc Vector (Thermo Fisher, 16146) to predict tumor burden from GLuc levels in blood (28). All cell lines were maintained as previously described (27). For primary cell culture, ascites-derived cells, splenocytes, or peripheral blood mononuclear cells (PBMCs) were incubated in RPMI-1640 (GIBCO) containing 10% FBS, 55 μM 2-β-mercaptoethanol, L-glutamine (HyClone), 10 mM HEPES, and 1% penicillin/streptomycin (GIBCO). All cells derived from primary tissues were filtered through a 70-μm cell strainer prior to coculture experiments. For IFN-γ treatment, mouse OC cell lines were treated with 500 pg/ml of mouse IFN-γ (PeproTech) for 48 h. All cell line cultures were PCR tested for mycoplasma routinely and prior to *in vivo* experiments. Cells were incubated at 37°C with 5% CO_2_ and used at low passage number for *in vivo* experiments.

To assess *in vitro* proliferation, the Incucyte® Live-Cell Analysis System was used to measure proliferation rates of ID8-p53−/− and ID8-p53−/−NLRC5+ cells. Cells were seeded at a low density in a 96-well plate (1,000 cells/well) and placed in an Incucyte® for incubation under standard growth conditions for live-cell analysis. Cell confluence was measured every 2 h until all cell lines reached full confluence. Raw data were normalized to align with the starting confluence at time zero of all biological replicates.

*Human cell lines:* A total of 10 different human OC cell lines derived from solid tumors (OVCAR-8, TOV-3041G, SK-OV-3), or from patient’s ascites (OV-90, OV-1946, PEO1, PEO4, A2780s, A2780cp, OVCA-420) (**Supplementary Table 1**) were used to screen for HLA I expression by flow cytometry and quantitative reverse transcription polymerase chain reaction (RT-qPCR). OV-1946 and TOV-3041G were cultured in ovarian surface epithelium (OSE, Wisent) media and 5% FBS. OVCA-420 were cultured in DMEM and 10% FBS. All other cell lines were cultured in RPMI-1640 (GIBCO) containing 10% FBS. Cell lines were plated at 0.5 × 10^6^/ml and cultured at 37°C in 5% CO_2_. For IFN-γ treatment, human cell lines were treated with 1 ng/ml human IFN-γ (PeproTech) for 72 h.

*Patient ovarian cancer samples:* RNA was extracted directly from frozen cell pellets derived from ascites samples obtained, with patient consent, from the Ottawa Ovarian Cancer Tissue Bank at The Ottawa Hospital (OHSN-REB Protocol #1999540-01H) (**Supplementary Table 2**).

### 2.2 Kaplan–Meier plot and correlation analysis

The Kaplan–Meier plotter for ovarian cancer (www.kmplot.com/ovar (29, 30)) was used as a prognostic tool, using microarray data derived from a large independent patient cohort of OC. The prognostic value for each gene of interest (*HLA-A*, *HLA-B*, *HLA-C*) resulted from a database that used gene expression and survival data from 1,287 OC patients as described by Gyorffy et al. (29). The serous histology subtype and P53 mutated status were applied to reach a total of n = 493 samples. Overall survival comparisons between low and high expressions were considered for these analyses. This platform was also used to assess the coefficient from correlation analysis using the Spearman test on different gene expressions compared with *NLRC5* expression (**Supplementary Table 3**).

For *NLRC5* prognostic analysis, TCGA database (www.proteinatlas.org (31)) was used to apply survival analysis to n = 373 OC samples and a cutoff of 2.01. The average expression level found in OC tumors was 1.57.

### 2.3 Single-cell RNA-seq analysis

Single-cell RNA-sequencing analysis of 16 ovarian tumors (15 of which were high-grade serous cancers) was performed by Hornburg et al. (24). Data were analyzed using the “Seurat” R-package (32, 33) with each individual tumor’s sample matrix divided into stroma, CD45+, and tumor cell files that we first made into Seurat objects with a minimum of 200 genes per cell and then merged using the “Seurat::merge()” function prior to processing. Each of the 16 individual samples was processed independently. Cells with high-percentage mitochondrial genes and low feature number were subset out, and then cell cycle genes were regressed out for each sample using “SCTransform”. “SCTransform” was also used to normalize the RNA matrices for each sample using regularized negative binomial regression. PCA was then applied to each individual sample, and UMAP embedding was calculated from the first 30 principal components. We also stash metadata such as immune phenotype and patient ID in each sample’s Seurat metadata slot. Differential gene expression was determined using the FindMarkers() “Seurat” function, and pathway analysis was done in the “fgsea” package (34) (**Supplementary Table 4**). The gene signature applied to mark cancer cells specifically was *ELF3*, *EPCAM*, *KRT19*, and *AMHR2*, and fibroblasts were marked by *COL1A1* and *COL1A2*. As reported by Hornburg et al. (24), immune cell infiltration was classified as infiltrated, excluded, and deserted, as determined by a combination of a machine learning transcriptional classifier (35) and CD8 IHC staining on samples.

### 2.4 Generation of NLRC5-overexpressing cell lines

NLRC5 open reading frames (ORF) were amplified from cDNA derived from a healthy C57BL/6 spleen. The genome-specific primer (GSP) for murine NLRC5 (mNLRC5-GSP 5′ CAACAGAGGTTCTTCTGAGCC) was used to make cDNA using SuperScript™ IV reverse transcriptase (Thermo Fisher). The full-length murine NLRC5 open reading frame (5.7 kb) was amplified from this cDNA by PCR (PCR Master Mix, Thermo Fisher) using the following primers: forward primer mNLRC5-InfF CTAGCCTCGAGGTTTGCCACCATGGACGCTGAGAGCATCC; reverse primer: mNLRC5-InfR TGCAGCCCGTAGTTTTCAAAGAGTCTGCTGGTCAGTG. Using In-Fusion cloning (Clontech® Laboratories), the gel-purified PCR product representing full-length murine NLRC5 was inserted into the pWPI (Addgene plasmid #12254) plasmid linearized with Pme1 (New England Biolabs). To express the same ORF in a vector without GFP expression (pLV), pWPI was modified to remove the IRES-eGFP cassette but maintain the context of the Pme1 cloning site for In-Fusion cloning (Clontech® Laboratories). Briefly, pWPI was digested with Pme1 and EcoR1 which removed the IRES-eGFP-WHV sequence. The digested product was gel purified (Illustra) following the manufacturer’s instructions. A PCR reaction (PCR Master Mix, Thermo Fisher) with 1 ng of pWPI was performed to amplify the WHV sequence and reinsert the Pme1 restriction site including 15 bases of sequence 3′ to the Pme1 site matching as found in pWPI, with the following primers: infDIG-F primer—CTAGCCTCGAGGTTTAAACTACGGGCTGCACTAGCTAGTCGAGCTCAACTTCG; infDIG-R primer—AAGCTTGAGCGAATTCCCG. This PCR product was inserted into the Pme1/EcoR1-digested pWPI to generate the vector pLV. ORFs were transferred from the pWPI constructs to the pLV vector (Pme1 cut) by In-Fusion cloning using PCR products amplified using the following primer sets: mNLRC5-InfF + mNLRC5-InfR, as described above. After a standard infusion reaction (Clontech® Laboratories), products were transformed into Stbl3 competent cells (Life Technologies). Colonies were grown up into minipreps (QIAprep Spin Miniprep Kit, QIAGEN) and the sequence was validated (10X Genomics, StemCore Laboratories, Ottawa Hospital). Lentiviral vectors were prepared by co-transfection of vector plasmids, with packaging plasmid pCMVR8.74 (Addgene plasmid #22036) and the ecotropic envelope expression plasmid, pCAG-Eco (Addgene plasmid #35617; RRID : Addgene_35617) into 293T cells as described previously (36).

ID8-p53−/− and STOSE cells were transduced with lentiviral particles from the pWPI+NLRC5 or pLV-NLRC5 lentiviral expressing vector. One week post lentiviral infection, cells were collected and sorted for GFP (pWPI) or MHC I (pLV) expression by using a Beckman MoFlo Astrios EQ flow cytometer.

### 2.5 Quantitative RT-PCR

Quantitative reverse transcription PCR (RT-qPCR) was performed using the ABI 7500 Fast (Applied Biosystems), and SsoAdvanced™ Universal SYBR® Green Supermix (Bio-Rad) was used to determine the relative gene expression. The mouse targeted genes included *Nlrc5*, *H2d*, *H2k*, *B2m*, *Tap1*, *Tap2*, *Lmp2*, *Lmp7*, and *Stat1*. *Ppia* and *Rlp0* were used as housekeeping control genes. The human targeted genes included *HLA-A, -B* and *-C, B2M, CD274,* and *NLRC5,* and *PPIA* was used as a housekeeping gene control. All primer sequences are presented in **Supplementary Table 5**. Total RNA was extracted and purified using Illustra RNAspin Mini Kit (GE Healthcare). NanoDrop™ One Microvolume UV-Vis Spectrophotometer (Thermo Scientific™) was used to assess the RNA quantity and quality. Complementary DNA (cDNA) was synthesized from 1 µg of RNA template using iScript™ Reverse Transcription Supermix (Bio-Rad) following the manufacturer’s instructions. The cycling conditions for the qPCR were as follows: 95°C for 5min, followed by 30 cycles of 15 s at 95°C and 60°C for 45 s. The mean of three technical replicates for each sample was used to represent data. The comparative method (ΔΔCT) was used to analyze the gene expression. The fold change in expression of each target gene was compared between the reference control and treated groups.

### 2.6 Mouse models and *in vivo* studies

All animal studies were performed under protocols approved by the Animal Care Committee at the University of Ottawa and conformed to the standards defined by the Canadian Council on Animal Care. All mice were 8–10-week-old female C57BL/6 obtained from The Jackson Laboratory (stock #000664). For intraperitoneal (IP) tumor development, mice were injected with 5 × 10^6^ cells in 100 μl PBS. To generate orthotopic tumors, 0.15 × 10^6^ cells were injected under the ovarian bursa (intrabursal injection, IB) of each ovary, for a total of 0.3 × 10^6^ cells/mouse. At collection time (day 51), orthotopic tumors and all macroscopic metastatic lesions in the peritoneal cavity were dissected and weighed. Mouse and mesenteric LNs weights were recorded, and cell number was determined for the peritoneal wash and mesenteric LNs.

*For prophylactic antitumoral study*: ID8p53−/− and ID8p53−/− NLRC5+ cells were collected using trypsin (0.05% trypsin, 0.53 mM EDTA, Thermo Fisher), counted and irradiated at 100 Gy (gamma irradiator). Cells were then counted and washed thrice with PBS before injecting into mice IP with 5 × 10^6^ cells in 100 μl PBS, as described elsewhere (27). Fourteen days after cell injection, mice were injected IP with 5 × 10^6^ cells viable parental ID8-p53−/− cells in 100 μl PBS and were monitored for survival until humane endpoint to assess if the irradiated cells could confer antitumoral protection.

*For subcutaneous tumor development:* 5 × 10^6^ cells in 100 μl PBS were injected in the right flank of each mouse. Tumor volume was measured every week using calipers and was calculated using the following formula: 1/2(length × width^2^), where width is the shortest diameter and length is the longer one. Mice were monitored for tumor burden and euthanized when the tumor showed signs of necrosis or when the tumor volume reached a maximum of 500 mm^3^.

### 2.7 Flow cytometry

Tumor-bearing mice were euthanized 5 days prior to reaching anticipated humane endpoint (day 51). Orthotopic tumors, mesenteric lymph nodes (mLNs), and peritoneal washes (PW) or ascites were collected, processed, and analyzed by flow cytometry. Tumors and tissues were dissected and reduced to small pieces to generate single-cell suspensions for extracellular and intracellular staining, as previously described (27). All samples were stained for cell viability discrimination and Fc-blocking of the CD16/CD32 (clone 2.4G2) antibody. Fully stained samples were fixed in 1% paraformaldehyde (PFA) and stored overnight at 4°C until acquisition in a BD FACSCelesta or Cytek™ Aurora Spectral flow cytometer. For intracellular staining, the Foxp3/Transcription Factor Staining Buffer Kit (eBioscience™) was used following the manufacturer’s protocol. See **Supplementary Table 6** for flow cytometry antibody details. All flow cytometry data were analyzed using FlowJo software (v10.8.1).

### 2.8 Immunohistochemistry and immunofluorescence

Hematoxylin and eosin staining was performed on 5-μm sections of formalin-fixed paraffin-embedded tumor tissue, as described elsewhere (27).

Immunofluorescence (IF) experiments were performed using 7-μm sections of tissue snap frozen in Tissue-Tek O.C.T. compound. Sections stored at −80°C were brought to room temperature and fixed using ice-cold methanol (for MHC I staining) for 20 minutes. Tissue sections were washed twice with PBS and blocked in 10% goat serum containing 0.01% Triton X-100 for 1 h at room temperature. Primary antibody for MHC I (Abcam, clone ER-HR 52) followed by secondary antibody of the appropriate specie (Invitrogen) were diluted in 10% goat serum in PBS. Slides were mounted with Immu-Mount (Thermo Fisher Scientific), cured overnight, and visualized on an Axioskop 2 MOT (Zeiss) microscope.

For NLRC5 IF assays, ID8-p53−/− and STOSE cells overexpressing or not NLRC5 were seeded (0.1 × 10^6^/ml) onto coverslips in a 12-well plate. As a positive control, cells were treated with 500 pg/ml mIFN-γ (PeproTech) and incubated for 48 h at 37°C in 5% CO_2_. After incubation, cells were fixed with 2% PFA in PBS and incubated at 37°C for 15 min. Cells were then permeabilized with 0.2% saponin (Sigma) in PBS for 15 min. Blocking solution containing 1% skim milk powder in PBS was applied to reduce false-positive signals. Cells were then incubated with the rat monoclonal primary antibody anti-human-NLRC5 (clone 3H8, Sigma) diluted in the blocking solution and left overnight at 4°C in a humid chamber. The next day after washing the cells with PBS, the secondary antibody was applied: anti-rat IgG-AF594 (clone MRG1-58, BioLegend) diluted in blocking solution and containing the dye Hoechst (Invitrogen). After 75 min of incubation in a humid chamber, the cells were washed with PBS and mounted using VECTASHIELD® Vibrance™ Antifade Mounting Medium (Vector Laboratories) for imaging. All images were captured using ZEISS ZEN Imaging Software.

### 2.9 LEGENDplex Bead-Based immunoassay

Ascites fluid was collected from the peritoneal cavity at endpoint from orthotopic tumor-bearing mice. All samples were centrifuged at 2,000 rpm for 15 min to collect supernatant that was immediately frozen and stored at −80°C until assay. Ascites supernatant was diluted 1:2 in assay buffer and assayed according to the manufacturer’s protocol for the LEGENDplex™ Mouse Anti-Virus Response Panel (13-plex) and the Cytokine Release Syndrome Panel with V-bottom Plate (BioLegend). Samples were acquired in duplicate the same day of staining on a CYTEK™ Aurora Spectral Flow Cytometer and analyzed using LEGENDplex Qognit software (BioLegend).

### 2.10 Ex-vivo PBMC activation

At 50 days after IP tumor cell injection, blood was obtained by saphenous bleeding and collected into heparin-coated capillary tubes (Microvette CB 300 Lh, Sarstedt). Blood was treated with ACK erythrocyte lysis buffer (Thermo Fisher), washed, and resuspended to determine the cell number. Peripheral blood mononuclear cells (PBMCs) were added at 1 × 10^6^ cells/ml to a precoated 12-well plate containing 2 μg of anti-CD3 (clone 2C11)/anti-CD28 (16–0281–85) (eBioscience) antibodies. Golgi-Plug and Golgi-Block agents (BD Biosciences) were added following manufacturer’s instructions to the cultures after 19 h of a total 24-h incubation time at 37°C. Cells were collected and processed for extracellular and intracellular flow cytometry analysis.

### 2.11 IFN-γ ELISpot

At 36 days after IP tumor cell injection, 100 μl–300 μl of blood was withdrawn from the saphenous vein and collected in a Microvette CB 300 LH (Cedarlane, Sarstedt, 16443100). Orthotopic tumor-bearing mice were challenged 5 days prior to anticipated humane endpoint with irradiated ID8-p53−/− or ID8-p53−/−NLRC5+ cells by IP injection. At endpoint, ascites and spleens were collected to generate a single-cell suspension as previously described (27). Samples were diluted in PBS with 2% FBS and centrifuged at 400g for 5 min. Supernatants were discarded, and cell pellets were treated with red blood cell lysis buffer (VWR) and resuspended in PBS+2% FBS, counted, and washed. IFN-γ 96-well ELISpot plates (Bio-Techne R&D Systems, ImmunoSpot C.T.L) were seeded with PMBCs (1 × 10^5^ cells), splenocytes (5 × 10^5^ cells), or ascites-derived cells (5 × 10^5^ cells) in RPMI complete media overnight at 37°C, in the presence of 5 μl/well of whole-cell lysates from irradiated ID8-p53−/−, ID8-p53−/−NLRC5+, or MC38 cells. Vesicular Stomatitis Virus (VSV)-N peptide (10 μM), complete media (negative control), or Phorbol 12-myristate 13-acetate (PMA) (50 ng/ml)/ionomycin (0.5 μg/ml; Sigma) (positive control) was used as control. The whole lysates were obtained by four cycles of freezing–thawing irradiated cells (100 Gy) by adding 10 × 10^6^ cells in 100 μl PBS. Lysates were centrifuged once to remove cell debris at 500g for 5 min. Supernatants were stored at −20°C until the day of the experiment. An ELISpot assay was performed following the manufacturer’s instructions with three technical replicates per biological replicate. IFN-γ secretion was quantified based on the number of spots by using an ImmunoSpot® Analyzer reader (Cellular Technology Limited, C.T.L.).

### 2.12 *In vivo* PD-L1 antibody-mediated blockade

ID8-p53−/− cells (5 × 10^6^/mouse) were injected IP and treated with anti-mouse PD-L1 antibody (GOLD, Clone 10F.9G2, Leinco Technologies Inc.) or rat IgG2b isotype control (GOLD, Clone 1-2, Leinco Technologies Inc.) starting approximately 25% into the expected time until endpoint (day 14). Mice were injected IP with 200 μg in 100 μl PBS/mouse daily for 5 days and then twice a week with a dose of 100 μg/mouse IP for 3 weeks, for a total of 11 doses. Mice were monitored until they reached humane endpoint to assess survival.

### 2.13 Infected cell vaccine generation

Rhabdoviruses Maraba MG1 and VSVΔ51-GFP were kindly provided by Dr. John Bell (Ottawa Hospital Research Institute). Viruses were propagated on Vero cells, purified by ultra-centrifugation, and quantified by the standard plaque assay, as previously described (37). Viral cytotoxicity was assessed on ID8 cells, and cell viability was carried out as described previously (38). Infected cell vaccines (ICV) were generated by lethally irradiating ID8-p53−/− cells at 100 Gy. Cells were then counted, washed, and resuspended in serum-free media at 2.5 × 10^6^ cells to infect them *in vitro* at a multiplicity of infection (MOI) of 10 pfu/cell to produce an ICV of 2.5 × 10^7^, and incubated for 2 h at 37°C with gentle rotation (37). Tumor-bearing mice received an IP injection of 100 μl of serum-free media containing 2.5 × 10^7^ of ICV from either MG1 or VSVΔ51-GFP, and serum-free media only as control. A total of three doses were given on days 9, 12, and 15 after tumor cell injection. Mice were monitored until they reached humane endpoint to assess survival.

### 2.14 Gaussia luciferase assay

To monitor tumor burden *in vivo*, blood samples were obtained weekly from the saphenous vein starting 1 week after ID8-p53−/−GLuc injection into C57BL/6 mice and until 5 weeks after tumor cell injection. Blood was centrifuged at 2,000 rpm for 15–20 min at room temperature, and plasma was recovered and stored at −80°C. Once thawed, 3 μl of every sample was diluted in PBS containing 0.1% BSA. Gaussia luciferase level in each sample was quantified by exposure to Coelenterazine-SOL (Nanolight) using a BioTek Synergy Mx plate reader and Gen5 2.07 Software.

### 2.15 Statistical analysis

All experiments were performed with a minimum of three biological replicates. All graphics and statistical analysis were generated using Prism 9.0 (GraphPad Software Inc.). Survival data were depicted in Kaplan–Meier plots, and statistical significance was calculated by log-rank (Mantel–Cox) tests. Student’s t-test was applied to compare two groups, one-way analysis of variance (ANOVA) was used to compare one variant in more than two groups, and two-way ANOVA followed by Tukey’s multiple comparisons test was used to compare tumor volumes between two groups over time. Data were considered statistically significant at *p* ≤ 0.05 (*p < 0.05; **p < 0.01; ***p < 0.001; ****p < 0.0001). Data are presented as the means ± SD or SEM as specified.

## 3 Results

### 3.1 High expression of NLRC5 correlates with prolonged survival of ovarian cancer patients

**Figure 1:**
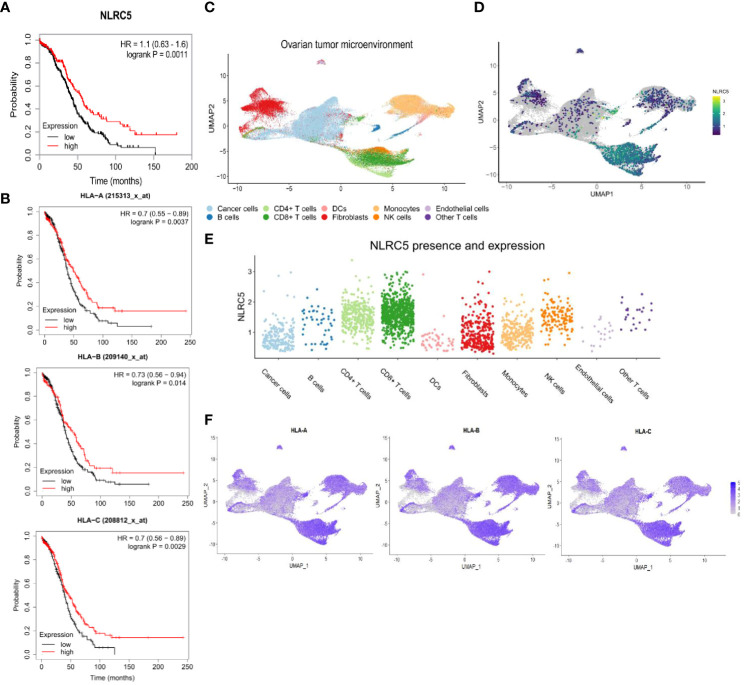
High *NLRC5* and HLA class I gene expressions are associated with favorable survival probability in OC patients. **(A)** Association of *NLRC5* expression with survival probability of OC patients. High *NLRC5* expression is associated with a 5-year survival of 45% compared with 26% of patients with tumors having a low *NLRC5* expression (*p* = 0.0011, best expression 2.01, median expression 1.57, N = 373 samples, I–IV disease stage). **(B)** Kaplan–Meier survival plots associated with expression of HLA class I molecules (HLA-A, B, C). Tumors possessing a serous histology and P53-mutated status were considered for these analyses (N = 493 samples (29)). **(C–F)** Analysis of single-cell RNA-sequencing data from 16 ovarian tumors (24). **(C)** UMAP analysis depicting cell clusters found in the TME of OC. **(D)** UMAP plots showing overall expression of *NLRC5*. **(E)** Relative expression of *NLRC5* in different cell types found in the TME of OC after filtering cells with no expression of *NLRC5* across all the populations. **(F)** UMAP plots showing overall expression of *HLA-A*, *-B*, and *-C*. Heatmaps display the level of expression in cell types as identified in **(C)**.

To test our hypothesis that NLRC5 expression in cancer cells results in better patient survival, we first probed the RNA-Seq TCGA and microarray database of human OC samples for expression of NLRC5 and its main target genes, including the classical HLA class I (*HLA-A-B-C*). This analysis demonstrated the role of NLRC5 as a positive prognostic indicator (p = 0.0011), wherein elevated expression correlated with a 45% 5-year survival rate, contrasting with the 26% survival rate linked to low or undetectable expression levels (**Figure 1A**). Likewise, ovarian tumors expressing classical *HLA-A*, *-B*, and -*C* were associated with increased survival (p = 0.0037, p = 0.014, and p = 0.0029, respectively) (**Figure 1B**). To investigate the cellular source of *NLRC5* expression in human OC, we used a high-resolution scRNA-seq library consisting of 16 ovarian tumors (24). UMAP clustering of these tumors identified 10 main cell populations, and, not surprisingly, the most abundant were cancer cells, myeloid cells, and fibroblasts (**Figure 1C**; **Supplementary Figure 1A**). *NLRC5* expression was found mostly in the immune compartment, specifically in T lymphocytes and natural killer (NK) cells, with few cancer cells displaying *NLRC5* expression (**Figures 1D, E**). Classical HLA class I haplotypes were expressed in almost all cellular components of the OC TME, but like *NLRC5*, there was less expression in the cancer cell population for all three HLA haplotypes (**Figure 1F**). The expression of other NLRC5 target genes was also examined (non-classical *HLA-E*, *-F*, *β2M*, *LMP2*, *TAP1*, **Supplementary Figure 1B**), and all of them were likewise expressed mainly in the immune compartment of the tumors. This emphasizes that genes associated with antigen presentation in transformed epithelial cells have less expression than in hematopoietic cells, which is likely exploited as a mechanism to maintain low expression of classical HLA I and reduced immune recognition (39) as observed in other solid cancer types (21, 40).

### 3.2 NLRC5 expression correlates with effector, cytotoxic, and pro-inflammatory immune markers in ovarian cancer

**Figure 2:**
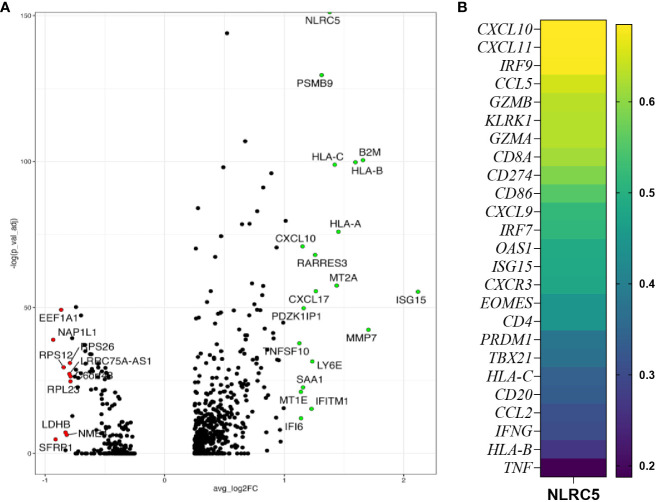
*NLRC5* expression correlates with immune-infiltrated TME. **(A)** Volcano plot showing the most differentially expressed genes in *NLRC5*+ cancer cells showing significantly upregulated (green) or downregulated (red) genes relative to cancer cells not expressing *NLRC5*. **(B)** Spearman correlation coefficient analysis of immune-related genes and *NLRC5* performed with data from the kmplot.com ovarian cancer database (29) (details in **Supplementary Table 3**).

To examine the possible role for NLRC5 in influencing immune cell infiltration, we performed a deeper analysis of scRNA-seq data (24) to discriminate *NLRC5* expression in the malignant cells or immune compartment (CD45+) according to tumor immune infiltration classification (**Supplementary Figure 2A**). Since *NLRC5* expression was found mostly in the immune compartment, unsurprisingly, CD45+ cells expressing *NLRC5* were most frequent in infiltrated tumors, followed by the immune excluded type. In cancer cells, the level of detection was overall very low but more prominent in the infiltrated tumors (~2%–5%) (**Supplementary Table 7**). We further investigated the transcriptional characteristics of *NLRC5+* cancer cells using the Gene Ontology pathways from GSEA, which showed overall increased expression of IFN, APM, and T-cell mediated cytotoxicity pathways (**Supplementary Figure 2B**). In addition to NLRC5’s known target genes that we can use as surrogates of NLRC5’s expression (*PSMB9*, *HLA-C*, *B2M*, *HLA-B*, *HLA-A*), we found several upregulated genes related to IFN response pathways such us *CXCL10*, *ISG15*, *MT2A*, *IFITM1*, and *IFIT6* (**Figure 2A**; **Supplementary Table 4**), suggesting a more pro-inflammatory environment under which NLRC5 upregulation could be triggered by the presence of IFN-γ, thereby fostering its increased production. To validate and complement these findings, we selected some specific genes related to antitumoral immunity and IFN responses (**Supplementary Table 3**), and applied Spearman correlation analysis (29). **Figure 2B** displays the strongest to the weakest Spearman correlation coefficients for genes that significantly correlated with *NLRC5* expression. Positive correlations were found not only between NLRC5 and APM and HLA I genes but also with other pathways known to actively participate in effector and cytotoxic functions during antitumoral responses and IFN signature in the TME (**Supplementary Table 3**). These analyses associate NLRC5 expression not only with antigen presentation molecules consistent with previous findings (21) but also with increased expression of markers of Th1 T-cell differentiation (*CD4*, *CXCR3*) and effector functions of NK and CD8+ T cells (*KLRK1*, *EOMES*, *PRDM1*, *TBX21*, *TNF*, *GZMA*, *GZMB*) as well as interferon-responsive genes such as *CXCL9*-, -*10*, -*11*, *IRF7*, *IRF9*, *CCL5*, *CD274*, *CD86*, *OAS1*, *ISG15*, and *IFNG*, possibly revealing a complex network regulated by NLRC5, beyond antigen presentation in OC.

### 3.3 MHC I downregulation by human OC cells is reversible under inflammatory conditions

**Figure 3:**
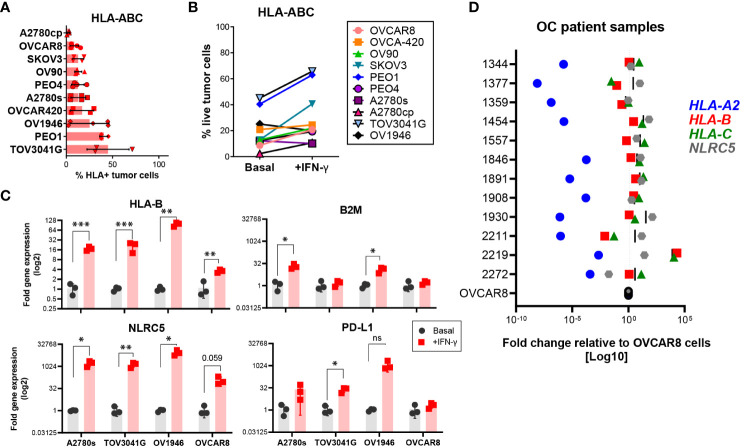
Most human OC cell lines express low levels of HLA I. HLA-ABC protein expression on ten different OC cell lines at **(A)** basal levels or **(B)** after IFN-γ treatment for 72 h, assessed by flow cytometry. Frequency of cells with detectable protein expression relative to all live cells. Bars represent three independent experiments, each with two to three technical replicates. Mean values and SEM error bars are shown. Cells were gated as singlet, viable cells. **(C)** A2780s, TOV3041G, OV1946, and OVCAR8 cell lines were assessed for expression of *HLA-B*, *B2M*, *NLRC5*, and *CD274* genes at basal (black) and after IFN-γ treatment (red) for 48 h. Data represent three independent experiments with three technical replicates. Significance was determined by unpaired t-test, *p < 0.05, **p < 0.01, ***p < 0.001. **(D)** OC ascites samples were assessed by qPCR for gene expression of *HLA-A2* (blue), *-B* (red), *-C* (green), and *NLRC5* (gray). Each dot represents the mean fold change (log 10 scale) of each sample relative to OVCAR8 cells used as control.

To identify appropriate models in which NLRC5 function could be explored, we screened 10 patient-derived OC cell lines derived from different histologic subtypes of OC (**Supplementary Table 1**). As shown in **Figure 3A**, less than half (~3%–45%) of cells in all tested cell lines had detectable HLA-ABC protein expression. Since MHC I downregulation can be a consequence of irreversible or “hard” HLA lesions including molecular alterations responsible for the loss of heterozygosity (40), we sought to determine if MHC I expression could be increased under inflammatory conditions. To this end, the same cell lines were treated with IFN-γ and protein and gene expressions for IFN-responsive genes, including *NLRC5*, *HLA-B*, *B2M*, and *PD-L1*, were assessed. As shown in **Figure 3B**, almost all tested cell lines had an IFN-γ-dependent increase in HLA-ABC expression; however, A2780s, OV1946, and OVCA-420 were refractory to this stimulation at the protein level. At the transcriptional level, *NLRC5* and *HLA-B* expression was increased in all studied cell lines in response to IFN-γ, indicating that these cells retain an intact APM machinery reversible under inflammatory conditions (**Figure 3C**).

To validate our findings, we assessed, at the transcriptional level, *NLRC5* and classic HLA I expression in cells from ascites (peritoneal fluid) from OC patient samples from our tumor bank (see **Supplementary Table 2**). Within each tumor, HLA haplotypes showed similar expression patterns in most of the analyzed samples (**Figure 3D**) relative to OVCAR8 cells, an OC cell line possessing low levels of HLA I expression. *HLA-A2* was very low in all samples whereas *HLA-B* and *-C* displayed comparable levels of expression, being very low in only 2/12 samples. *NLRC5* expression was comparable with *HLA-B* and *-C* levels with the exemption of 3/12 samples where its expression was slightly higher. These findings confirm the poor expression of NLRC5 and classic HLA I molecules in ascites-derived OC cells, validating the low levels of expression found in primary tumors (**Figures 1D–F**).

Collectively, these findings validate the bioinformatics analysis of the scRNA-seq data, showing low levels of NLRC5 expression in most OC samples. Most of the OC cell lines possess “reversible” HLA lesions which allow for induction under inflammatory conditions.

### 3.4 NLRC5 overexpression in ovarian cancer cells increases MHC I and antigen processing and presentation machinery expression

**Figure 4:**
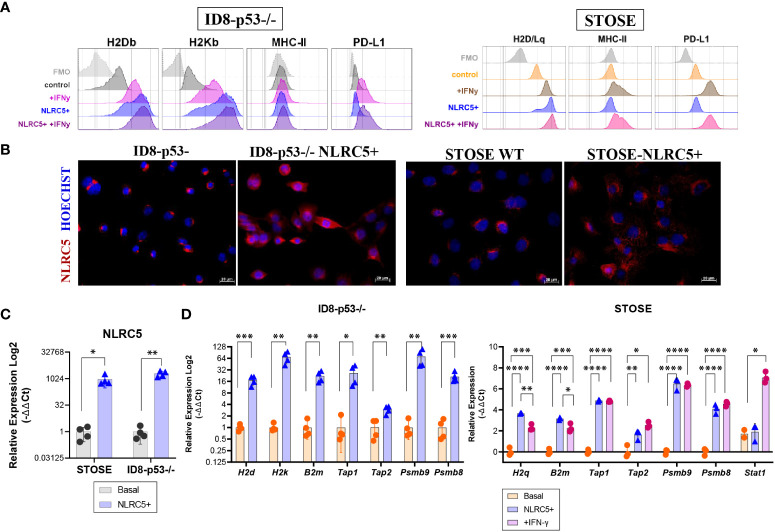
NLRC5 overexpression in OC cells rescues MHC I expression. **(A)** Histograms depicting the mean fluorescence intensity (MFI) of H2Db, H2Kb (H2D/Lq for STOSE), MHC-II, and PD-L1 protein expression assessed by flow cytometry on ID8-p53 − */* − (left) or STOSE (right) cell lines overexpressing or not NLRC5 under basal conditions or in the presence of IFN-γ for 72 h. Histograms are representative of three independent experiments with three technical replicates. Cells were gated as singlet, viable cells, and fluorescence minus one (FMOs) are depicted in grey. **(B)** Immunofluorescence of ID8-p53−/− or STOSE cells displaying NLRC5 expression (red) at basal (middle panel) or after NLRC5 overexpression (right panel). Hoechst dye was used to visualize the nuclei. Scale bars = 20 μm. Images representative of three independent experiments. Relative expression assessed by qPCR for **(C)** *Nlrc5*, and its target genes **(D)** *H2k*, *H2d*, *B2m*, *Tap1*, *Tap2*, *Lmp2*, and *Lmp7* in NLRC5-overexpressing relative to untreated ID8-p53−/− cells or STOSE cells as indicated. STOSE treated with IFN-γ for 72 h were used as control for all genes. The relative expression was normalized to *Rpl0* and *Ppia* genes. T-test or one-way ANOVA followed by Tukey’s *post-hoc* test was used to identify differences between groups, *p < 0.05, **p < 0.01, ***p < 0.001, ****p < 0.0001. Error bars indicate ± SD. N = 3–4 biological replicates for each cell line.

Previous studies have suggested that NLRC5 can regulate MHC I-dependent antitumoral responses (20, 21), and our findings associate NLRC5 expression in the TME with increased immune infiltration which could impact therapeutic outcomes (**Figure 2**; **Supplementary Figure 2A**). To investigate the therapeutic potential of NLRC5 in OC, we employed the ID8-*Trp53^−/−^* (hereafter ID8-p53−/−) cell line, which possesses “soft/reversible” MHC I lesions that can be overcome by IFN-γ treatment (**Figure 4A**, left panel) and that generate “cold” tumors (27). For comparison, we used the STOSE cell line which inherently expresses MHC I that can be further upregulated by IFN-γ (**Figure 4A**, right panel) and creates “hot” tumors (27). ID8-p53−/− and STOSE cells overexpressing NLRC5 were generated by lentiviral transduction, and protein expression was confirmed by immunofluorescence (**Figure 4B**). NLRC5-mediated MHC I gene expression requires an intact nuclear localization signal and nuclear distribution (41). However, in both control cell lines, endogenous NLRC5 distribution was mostly confined to the peri-nuclear region, which may suggest a decreased functionality for MHC I and APM gene induction, especially in the ID8-p53−/− cell line. NLRC5 overexpression did not alter the basal expression of MHC II or PD-L1, but the MHC I haplotypes H2Db and H2Kb (ID8) and H2D/Lq (STOSE) were significantly increased in NLRC5-overexpressing cells, even beyond levels stimulated by IFN-γ alone (**Supplementary Figure 3**). To further characterize NLRC5+ OC cell lines, we assessed the expression of other NLRC5 target genes by qPCR, such as *H2k*, *H2d*, *H2q*, *B2m*, *Tap1*, *Tap2*, *Lmp2*, and *Lmp7*. *Nlrc5* was strongly induced in both NLRC5+ cell lines (**Figure 4C**) as well as NLRC5 target genes which were also significantly induced by IFN-γ in STOSE cell lines (**Figure 4D**).

Overall, these results validate the overexpression and functionality of NLRC5 in both ID8-p53−/− and STOSE cell lines. By overexpressing NLRC5, MHC I expression can be recovered in ID8-p53−/− cells, which could have an impact on tumor immunogenicity *in vivo*. We further subsequently used the ID8-p53−/− model to assess whether NLRC5 overexpression could modulate tumor immunogenicity in “cold” tumors, which represent around 60% of human OC (24).

### 3.5 NLRC5 overexpression delays subcutaneous ovarian cancer development and modifies the tumor immune composition

**Figure 5:**
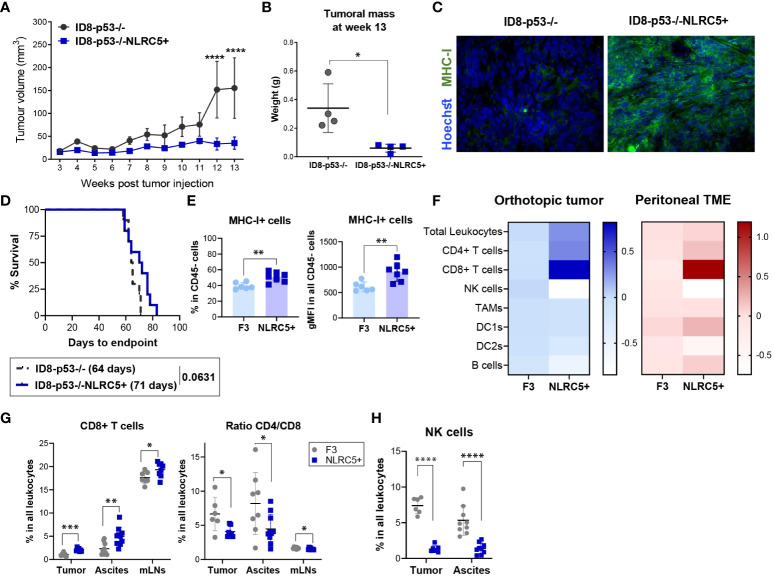
NLRC5 overexpression delays OC tumor growth and shapes the orthotopic TME. **(A)** Tumor volume over time after injection of 5 × 10^6^ of ID8-p53−/− or ID8-p53−/−NLRC5+ cells subcutaneously (SC). Tumor volume differences were compared using two-way ANOVA followed by Tukey’s *post-hoc* test, ****p < 0.0001 (n = 5). **(B)** Tumoral mass measured 13 weeks after SC tumor cell injection. Significance was determined by unpaired t-test, *p < 0.05 (n = 4). **(C)** Immunofluorescence of ID8-p53−/− (left) or ID8-p53−/−NLRC5+ (right) SC tumors displaying MHC I expression (green) at week 13 after tumor cell injection. Hoechst dye was used to visualize the nuclei. Scale bars = 50μm. **(D)** Survival Kaplan–Meier plots of ID8-p53−/− or ID8-p53−/−NLRC5+ tumor-bearing mice injected with 1.5 × 10^5^ cells under each ovarian bursa (n = 10). Data are representative of two independent experiments with similar results. Median survival is depicted in parentheses. **(E–H)** Orthotopic tumors, ascites, and mesenteric lymph nodes (mLNs) were collected at day 51 to assess the immune composition by flow cytometry (**Supplementary Figure 4A**). F3 = ID8-p53−/−, C5 = ID8-p53−/−NLRC5+. **(E)** Percentage of cells expressing MHC I of all CD45− cells in the tumor (left) and level of expression (right). **(F)** Heatmap showing average fold changes relative to parental tumors of main immune cell populations within **(E)** orthotopic tumors (blue) or ascitic TME (red) based on frequency in the CD45+ population (zero scale being no change). **(G)** CD8+ T cells, ratio CD4/CD8 T cells, and **(H)** NK cell frequencies in all leukocytes in the tumors, ascites, and mLNs. Each dot represents a biological replicate. Data present n = 6–7 orthotopic tumors/cell type, n = 9–10 ascites/cell type, and n = 9 mLNs/cell type. Significance was determined by unpaired t-test, *p < 0.05, **p < 0.01, ***p < 0.001, ****p < 0.0001.

To evaluate if the rescue of MHC I expression triggered by NLRC5 overexpression could influence tumor development through the MHC-I-peptide-CTL axis, *in vivo* tumor growth was assessed in different TMEs, by subcutaneous (SC), orthotopic (under the ovarian bursa, IB), or intraperitoneal (IP) injections of ID8-p53−/− and ID8-p53−/−NLRC5+ cells into C57BL/6 mice. First, no differences in the proliferation rate *in vitro* were noted (**Supplementary Figure 4A**). However, when injected under the skin a significant delay in tumor growth and reduced tumoral mass was found with SC tumors (**Figures 5A, B**; **Supplementary Figure 4B**). MHC I expression was confirmed at endpoint by immunofluorescence of tumor samples (**Figure 5C**), validating NLRC5-driven MHC I expression during tumor growth. Unfortunately, when tumor cells were injected orthotopically or in the peritoneal cavity, no differences were found in tumor development (**Figure 5D**; **Supplementary Figures 4C, D**), although there was a trend (p = 0.06) for longer survival in mice with NLRC5+ IP tumors. These findings emphasize how a more restricted TME (under the skin) allows for the generation of significant antitumoral responses by the increased immunogenicity of the ID8-p53−/−NLRC5+ cells, similar to that previously observed with the B16-F10 melanoma model (20). In contrast, the peritoneal cavity is recognized for its heightened immunosuppressive qualities (42) triggering distinct neoplastic characteristics in ovarian cancer cells, such as anoikis resistance thereby exerting a significant influence on tumor progression (43).

Despite not observing an impact on survival within a biologically pertinent setting like the peritoneal cavity, we endeavored to explore whether increased NLRC5 expression could affect the immune composition of orthotopically located ID8 p53−/− tumors. This inquiry aimed to shed light on the underlying reasons for NLRC5’s inability to confer antitumor protection. Mice were euthanized at day 51 (advanced-stage disease) to assess the main immune subsets infiltrating tumors, mesenteric LNs (mLNs), and the peritoneal cavity by flow cytometry (**Supplementary Figure 5A**). At the time of collection, we noted a significant decrease in the metastatic burden of ID8-p53−/−NLRC5+ tumor-bearing mice which correlated with a significant decrease in cell number found in the peritoneal cavity (**Supplementary Figure 4E**). We then investigated whether MHC I expression was maintained through the progression of advanced disease, akin to what was observed in the development of SC tumors. MHC I was significantly higher in NLRC5+ tumors as shown by the frequencies of MHC I+CD45− cells (~40% of control vs. ~60% of NLRC5+) as well as the total expression of MHC I (**Figure 5E**). In contrast, the CD45− fraction found in the ascites, which represents the surrounding TME and local site of metastasis of OC (44), did not display significant differences in MHC I expression (**Supplementary Figure 5B**) potentially revealing a naturally occurring MHC I downregulation or loss of expression in metastatic cells in this model. **Figure 5F** summarizes the main immune populations identified in both TMEs with similar significant NLRC5-related changes found in both TMEs. The overall leukocytic infiltration in the primary tumors overexpressing NLRC5 was also significantly increased but not in the ascites (**Supplementary Figure 5C**). Interestingly, there was a considerable increase in CD8+ T cell infiltration in all three organs (tumor, ascites, and mLNs) along with a decreased ratio of CD4/CD8 T cells in NLRC5+ tumor-bearing mice, as shown in **Figure 5G**. In contrast, the total frequencies of NK cells were dramatically decreased in both the tumor and ascites, pointing to a negative impact on NK activity driven by NLRC5 or MHC I. No significant differences were observed for other immune subsets except for the DC2-like population (CD11c+CD11b+CD3−) in the ascites (**Supplementary Figures 5D, E**).

Taken together, these results indicate that NLRC5-triggered MHC I expression raises the overall presence of immune cells in the tumor. This notably impacts the proportion of T cells, specifically enhancing the infiltration of CD8+ T cells, while concurrently reducing the recruitment of NK cells in both the tumor and ascites.

### 3.6 NLRC5 overexpressing OC cells generate a less immunosuppressive TME

**Figure 6:**
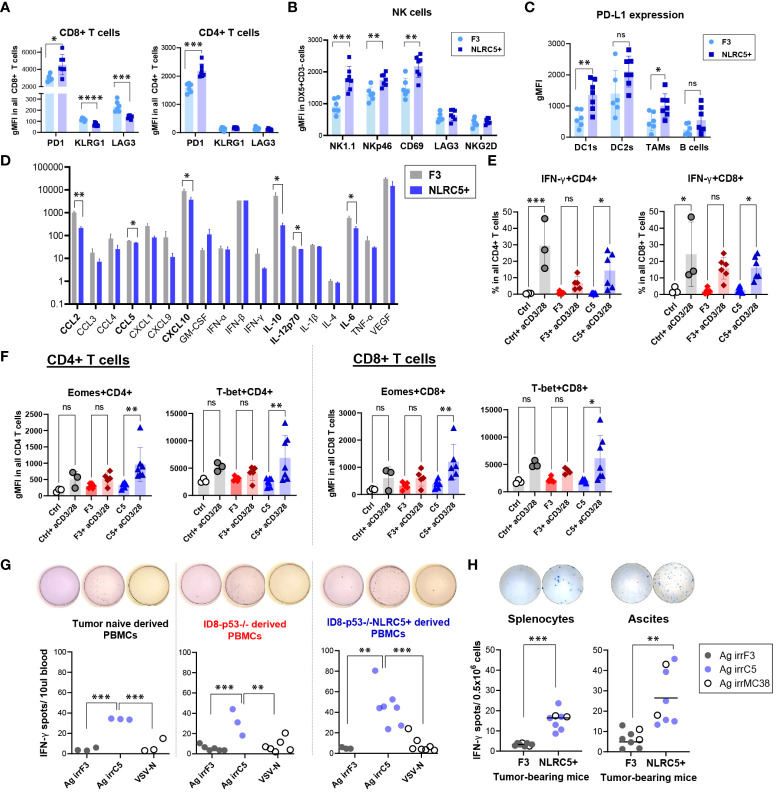
CD8+ T cells from NLRC5+ tumor-bearing mice possess a greater cytotoxic capability. **(A–C)** Phenotypic characterization of immune subsets from orthotopic tumor-bearing mice 51 days after tumor cell injection (summarized in **Figure 5F**), assessed by flow cytometry. Geometric mean fluorescence intensity (gMFI) depicting expression of **(A)** PD1, KLRG1, and LAG3 on CD8+ and CD4+ T cells; **(B)** NK1.1, NKp46, CD69, LAG3, and NKG2D expression on DX5+CD3- NK cells; and **(C)** PD-L1 expression in DC1s, DC2s, TAMs, and B cells in the orthotopic tumors. Significance was determined by unpaired t-test, ns, not significant, *p < 0.05, **p < 0.01, ***p < 0.001, ****p < 0.0001. **(D)** Histogram depicting the relative concentrations (pg/ml) of cytokines and chemokines in the ascites fluid from orthotopic tumor-bearing mice at endpoint. N = 4–9 biological replicates per tumor type. F3 = ID8-p53–/–, NLRC5+ = ID8-p53–/–NLRC5+. Significance was determined by unpaired t-test, *p < 0.05, **p < 0.01. **(E, F)** PBMCs from parental (red) and NLRC5+ (blue) tumor-bearing mice and tumor-naïve mice (gray) were stimulated ex-vivo for 24 h in the presence of anti-CD3/CD28 antibodies and analyzed for transcription factors, activation, and cytotoxic markers as depicted in **Supplementary Figure 7A**. **(E)** IFN-γ frequencies in CD4+ (left panel) and CD8+ (right panel) T cells, and **(F)** gMFI depicting total expression of Eomes and T-bet in PBMCs from n = 3 tumor-naïve mice, n = 6 ID8-p53−/− and n = 7 ID8-p53−/−NLRC5+ tumor-bearing mice. Significance was determined by one-way ANOVA, followed by Tukey’s *post-hoc* test, ns = not significant, *p < 0.05, **p < 0.01, ***p < 0.001. **(G, H)** Tumor-naïve or tumor-bearing mice were challenged at day 30 with an infected cell vaccine consisting of ID8-p53−/− or ID8-p53−/−NLRC5+ cells irradiated at 100 Gy and infected *ex vivo* with VSVΔ51. Six days later, PBMCs were obtained from the saphenous vein and assessed for IFN-γ ELISpot in the presence of whole cell lysates: Ag irrF3 = from irradiated ID8-p53−/−; Ag irrC5 = from irradiated ID8-p53−/−NLRC5+, VSV-N peptide was used as a specific control for VSV infection (**Supplementary Figure 7H**). Each dot is representative of one biological replicate. Significance determined by one-way ANOVA, and Tukey’s *post-hoc* test, **p < 0.01, ***p < 0.001. **(H)** Orthotopic tumor-bearing mice were challenged 5 days prior to euthanization by IP injection of ID8-p53−/− or ID8-p53−/−NLRC5+ cells irradiated at 100 Gy. At endpoint, splenocytes and ascites cells were collected and assessed overnight for IFN-γ ELISpot, as described in **(G)** Whole-cell lysates, Ag irrF3, Ag irrC5, and Ag irrMC38 = whole lysate from irradiated MC38 cells, were used to determine cross-reactivity between peritoneal carcinomatosis models. Each dot is representative of one biological replicate. Significance determined by one-way ANOVA, and Tukey’s *post-hoc* test, **p < 0.01, ***p < 0.001.

To comprehensively dissect the impact of NLRC5 overexpression in the TME on T and NK lymphocytes present in both the tumor and ascites, we further examined their functional characteristics by assessing the expression of activation/exhaustion markers (CD127, CD25, Ly6C, PD-1, KLRG1, LAG3, CD69, NKG2D) as summarized in **Supplementary Figure 5A**. The most significant changes were found in the tumor-infiltrating lymphocytes. First, almost all CD8+ T cells were PD1+ but displayed significantly less KLRG1 and LAG3 expression (**Figure 6A**; **Supplementary Figures 6A**). The proportion of CD127+CD8+ T cells was also significantly decreased in NLRC5+ tumors, but no significant changes were found for the activation markers CD25 and Ly6C (**Supplementary Figure 6B**, top panel). Similar outcomes were found for the PD1+CD4+ T cell subsets (**Figure 6A**) without changes in the expression of activation markers (**Supplementary Figure 6B**, bottom panel). T lymphocytes infiltrating the ascites were less impacted by NLRC5 overexpression than those found in the tumor (**Supplementary Figure 6C**). Despite being less frequently found in the tumor and ascites (**Figure 5H**), NK cells in tumors displayed significantly increased expression of activating markers such as NK1.1, NKp46, and CD69, but not LAG3 nor NKG2D (**Figure 6B**). Ascites-derived NK cells showed no difference in activation but had a slightly increased expression of NKG2D (**Supplementary Figure 6D**).

PD-L1 expression was similarly determined on different immune subsets. Some antigen-presenting cells such as CD11c+ DC1s and F4/80+ TAMs (**Figure 6C**), but not DC2s nor B cells, displayed significantly increased PD-L1 expression, highlighting a local response induced by NLRC5 overexpression which was absent in the ascites (**Supplementary Figure 6E**). Unexpectedly, when analyzing the CD45− portion, the proportion of PD-L1+ cells was significantly decreased in non-immune cells found in the NLRC5+ tumors compared with control (~25% vs. ~35%, respectively), but the overall level of expression of PD-L1 in this cellular compartment was similar (**Supplementary Figure 6F**). Altogether, these phenotypic findings point toward a potentially increased T-cell reactivity inside the tumor bed and therefore IFN-γ production in the orthotopic TME, which seems to be lost or decreased in the ascites.

To complete the analysis of the local TME, cytokine and chemokine profiling was performed on the ascites supernatant derived from tumor-bearing mice at endpoint when this peritoneal fluid is more accessible for collection. Six out of 18 chemokines/cytokines assessed were significantly decreased in the ascites from NLRC5+ tumor-bearing mice (**Figure 6D**), including IL-6, IL-10, IL-12, CXCL10, CCL5, and to a greater extent, CCL2. Decreased IL-10 and CCL2 suggest a less immunosuppressive TME and correlate with the immune composition of the ascites that showed a decreased recruitment of tolerogenic DC2s and NK cells (**Figure 5H**; **Supplementary Figure 5E**).

In line with these findings, and despite showing a similar rate of tumor development, NLRC5 overexpression in the ID8-p53−/− model shapes the orthotopic and ascitic TME, increasing T-cell activation as shown by PD-1 and PD-L1 expression in T lymphocytes and APCs, respectively. NLRC5 overexpression also decreases the establishment of an immunosuppressive ascitic TME. However, the attempt to stimulate noteworthy antitumoral immune response through priming with NLRC5 revealed limitations in generating substantial levels of immune activity against tumors.

### 3.7 NLRC5-overexpressing ID8-p53−/− tumors increase systemic T-cell reactivity toward autologous tumor-associated antigens

Since ascites originating from ID8-p53−/−NLRC5+ tumors exhibit reduced immunosuppressive effects, we aimed to explore whether the effector functions of circulating T cells mirrors an enhanced responsiveness. PBMCs were isolated at day 50 from IP tumor-bearing mice and activated with anti-CD3/CD28 for 24 h, then analyzed by flow cytometry to determine the extent of T-cell activation, and expression of key transcription factors and effector molecules (**Supplementary Figure 7A**). Both CD4+ and CD8+ T cell compartments were efficiently activated as tumor-naïve (Ctrl) PBMCs and displayed similar cell frequencies (**Supplementary Figures 7B, C**, respectively). Nonetheless, IFN-γ was significantly secreted by T cells derived from ID8-p53−/−NLRC5+ tumor-bearing mice compared with the mice bearing parental tumors (F3) (**Figure 6E**), suggesting that the immunosuppressive TME in the ascites has a systemic effect on T-cell functionality.

Eomesodermin (Eomes) and T-box expressed in T cells (T-bet) are two master regulators of T-cell effector functions, including IFN-γ production and cytotoxicity (45). Total expression of Eomes and T-bet transcription factors were significantly increased only in the T cells derived from NLRC5+ (C5) tumor-bearing mice (**Figure 6F**; **Supplementary Figure 7D**) underlining an increased T-cell effector potency, absent in T cells derived from the parental ID8-p53−/− (F3) tumor-bearing mice. Comparable outcomes were also noted among PD-1+ and CXCR3+ T cells (**Supplementary Figures 7F, G**, respectively) extracted from mice bearing NLRC5+ tumors, underscoring once more their heightened potential to carry out effector functions proficiently.

To complete the assessment of T-cell functionality, we investigated T-cell reactivity toward autologous TAAs by IFN-γ ELISpot. As previously shown (27), the ID8-p53−/− model generates a “cold” TME lacking T-cell “priming” (**Figures 5F**, **6A**). To circumvent the absence of T-cell activation or “priming”, tumor-bearing mice were pre-immunized with an infected cell vaccine (ICV) consisting of irradiated ID8-p53−/− NLRC5+/− cells infected *ex vivo* with the oncolytic virus VSVΔ51 and compared with the parental control mice (**Supplementary Figure 7H**). The irradiation and infection enable the ICV to present a multitude of TAAs in the context of a robust oncolytic virus infection, a combination that leads to potent immune stimulation *in vivo* (46). As shown in **Figure 6G**, when reexposed to exogenous TAAs derived from ID8-p53−/−NLRC5+ cells (Ag irrC5), PBMCs derived from parental or NLRC5+ tumor-bearing mice secreted significantly more IFN-γ compared with PBMCs reacting toward Ag irrF3 or the control VSV-N peptide. Remarkably, even in tumor-naïve mice, PBMCs recognized and secreted IFN-γ only in the presence of TAAs derived from NLRC5+ tumor cells, potentially underlining overproduction/presentation of self-peptides recognized by circulating T cells (**Figure 6G**, left panel). Moreover, PBMCs derived from ID8-p53−/− mice were also more reactive toward Ag irrC5 but not from parental Ag irrF3 (**Figure 6G**, middle panel). IFN-γ production was also significantly enhanced in splenocytes and ascites derived from ID8-p53−/−NLRC5+ tumor-bearing mice even in the presence of Ag irrMC38 (derived from MC38), once more emphasizing autologous reactivity against self-shared peptides also generated by this colorectal cancer cell line (**Figure 6H**).

Collectively, NLRC5 overexpression in ID8-p53−/− tumor cells increased reactivity of T cells but also amplified T-cell recognition against autologous self-peptides shared by cancer cells originating from the peritoneal cavity. Circulating T cells displayed a stronger activation profile compared with those from parental tumors, and importantly, IFN-γ production was significantly induced when PBMCs, splenocytes, and ascites-derived cells from ID8-p53−/−NLRC5+ tumor-bearing mice were exposed to TAAs from NLRC5+ tumor cells, emphasizing a greater ability to increase the production and recognition of naturally occurring TAAs.

### 3.8 NLRC5 overexpression in ovarian cancer cells improves efficacy of an infected cell vaccine

Considering our findings, NLRC5 overexpression remodeled the TME of the ID8-p53−/− model rendering it potentially “hotter” and increasing TAA generation and presentation. Next, we sought to examine if the ID8-p53−/−NLRC5+ cells could be exploited as a prophylactic cellular vaccine to confer antitumoral protection against parental tumor development. However, prophylactic cellular vaccination with irradiated ID8s with basal or NLRC5 overexpression did not alter survival of mice upon challenge with parental cells, underlying the absence of immunogenicity of this tumor model (**Supplementary Figure 8A**).

Recent evidence has indicated that tumors could be more responsive to ICIs when expressing NLRC5 and APM proteins in the tumor niche (47, 48). To this end, ID8-p53−/− or ID8-p53−/−NLRC5+ cells were injected IP into syngeneic mice and treated with anti-PD-L1 or isotype control for an extended period of time (**Supplementary Figure 8B**). PD-L1 blockage did not prolong survival in the ID8-p53−/− model (49) even when NLRC5 was overexpressed, as the median survivals were similar (**Supplementary Figure 8C**). Nonetheless, the proportion of long-term responders increased in mice bearing NLRC5+ tumors, as at 106 days, 40% of mice were still alive compared with 20% in the group with ID8-p53−/− tumors (**Supplementary Figure 8D**). This observation challenges any potential benefit driven by NLRC5 overexpression in this indolent tumor model but shows evidence of an increased capability to respond to anti-PDL1 treatment in some mice.

**Figure 7:**
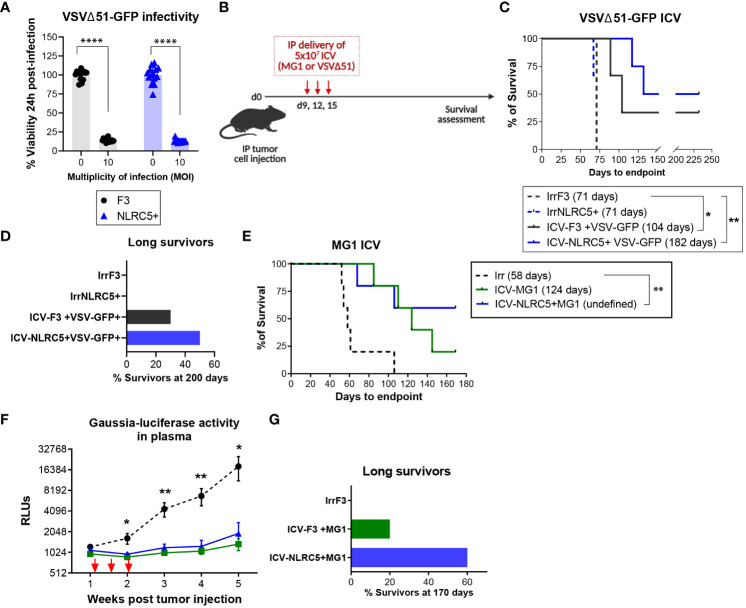
NLRC5 overexpression increases efficacy of an infected cellular vaccine. **(A)** Cell viability 24 h after *in vitro* infection with VSVΔ51 at a MOI of 10, assessed by AlamarBlue. There were 14 replicates assessed per condition. Data representative of two independent experiments. Significance was determined by unpaired t-test, ****p < 0.0001. **(B)** Therapeutic ICV regimen. Mice were injected IP with parental ID8-p53−/− cells **(C)** or ID8-p53−/−GLuc cells **(E)** and received an ICV dose on days 9, 12, and 15, to assess survival until endpoint. **(C)** Survival Kaplan−Meier plots and **(D)** percentage of survivors at day 200 of ID8-p53−/− tumor-bearing mice treated with ICV-VSVΔ51 as shown in **(B)**. N = 8 mice/treatment. Median survival depicted in parenthesis. Log-rank (Mantel–Cox) *p < 0.05, **p < 0.01. **(E–G)** ID8-p53−/−GLuc tumor-bearing mice treated with ICV-MG1 as shown in **(B)**. **(E)** Survival Kaplan–Meier plots and, **(G)** percentage of survivors at day 170 after treatment as depicted in **(B)**. Median survival depicted in parenthesis. Log-rank (Mantel–Cox) **p < 0.01. **(F)** Gaussia luciferase activity in plasma from tumor-bearing mice. Differences in relative luminescence units (RLU) were assessed using two-way ANOVA followed by Tukey’s *post-hoc* test, *p < 0.05, **p < 0.01. N = 5 mice/treatment.

Finally, we sought to investigate if NLRC5 overexpression in an ICV could increase antitumoral protection, given that mice bearing NLRC5+ tumors generated T cells with greater activation and recognition of autologous TAAs and that the infection triggered by an oncolytic virus would render the cellular vaccine more immunogenic (**Figure 6G**). The rate of infectivity at a MOI of 10 *in vitro* was similar between cell lines regardless of NLRC5 expression (**Figure 7A**), suggesting that NLRC5 does not interfere with viral infection in this model. Mice were injected with parental ID8-p53−/− or ID8-p53−/− G-Luc tumor cells to allow for monitoring tumor progression and response to treatment (28). Mice received three doses of an ICV, as shown in **Figure 7B**. Two different oncolytic Rhabdovirus platforms were used, VSVΔ51 or Maraba MG1, as they can productively infect target cells and increase TAA release (50, 51). As shown in **Figures 7C–G**, three doses of an ICV consisting of VSVΔ51 or MG1 conferred significant antitumoral protection against ID8-p53−/− tumors, which was evident even during early tumor growth as shown by Gaussia-luciferase activity (**Figure 7F**). Remarkably, when the ICV overexpressed NLRC5, the proportion of survivors was greater with longer efficacy over time (**Figure 7G**). Thus, NLRC5 overexpression in an ICV can enhance antitumoral protection by increasing TAA recognition, T-cell activity, and overall decreased immunosuppression, acting in synergy with an oncolytic cellular vaccine.

## 4 Discussion

Tumor antigen presentation is a key factor to trigger a productive CTL recognition and tumor cell elimination. However, antigen presentation is downregulated in around 60% of OCs, playing a significant role in cancer immune evasion. Therefore, the discovery of new strategies enabling increased TAA presentation and MHC I expression is imperative. The present study is the first to investigate the therapeutic potential of MHC I-driven expression by NLRC5 overexpression in OC. We found that, overall, OC displays a very weak expression of NLRC5 and its main target genes, which could be one of the mechanisms maintained by neoplastic cells to avoid antitumor immunity (21). Moreover, NLRC5 possesses a prognostic value when highly expressed in OC tumors. Lack of NLRC5’s expression in OC cells may participate in the generation of a “cold” and poorly immunogenic TME by affecting the presentation of TAAs to the immune compartment (52, 53). Similar to many other cancer types, classic MHC I expression in OC correlates with better prognosis (21, 54). NLRC5 expression was mainly found in the immune compartment of OC acting as a positive prognostic factor for high-grade OC patients and overall survival. Human OC cells expressing NLRC5 display a gene signature positively correlating with immune and IFN-related genes potentially featuring ongoing natural antitumoral immunity, as has been noted in melanoma and Tasmanian devil transmissible cancer cells (55, 56).

By inducing NLRC5 overexpression in a “cold” OC murine model ID8-p53−/− which naturally possess very low MHC I expression *in vitro* and *in vivo (* 27), we demonstrated that MHC I haplotype expression can be recovered as well as expression of other APM genes such as *B2m*, *Tap1/2*, and *Lmp2/7*. NLRC5 overexpression was able to delay ID8-p53−/− tumor growth when administered subcutaneously but did not have a significant impact on tumor development in the peritoneal cavity. This finding may be explained by the greater capability of OC cells to increase their metastatic potential by adhesion to the peritoneum, and survival in a non-permissive TME (57, 58). This peritoneal environment provides easy access to adipose tissue modifying cancer cell metabolism, and hypoxic conditions, rendering the TME more complex to achieve antitumoral immunity (59).

Nevertheless, orthotopic NLRC5-overexpressing tumors contained significantly more leukocytes and CD8+ T cells, similar to the ascites and mesenteric LNs. Importantly, these CD8+ T cells displayed a more prominent activation state but less exhausted phenotype by decreased expression of KLRG1 and LAG3, presumably induced by a greater recognition of TAAs in the tumor niche. NK cells were dramatically decreased in both the orthotopic tumor bed and the ascites, presumably through the negative regulation induced by classic MHC I molecules. Circulating T cells derived from NLRC5-overexpressing tumor-bearing mice showed a greater ability to be activated, producing higher amounts of IFN-γ and displaying higher expression of T-bet and Eomes transcription factors which are known to cooperate to regulate CD8+ T cell effector function (60). Remarkably, when exposed to whole NLRC5+ cell lysates, PBMCs, splenocytes, and ascites cells obtained from NLRC5-overexpressing tumors were capable of strongly producing IFN-γ; even PBMCs from tumor-naïve mice showed T-cell activation potentially detecting naturally occurring self-antigens, which could be reflecting low-affinity T-cell clones (61).

Other significant changes were found in the ascites chemokine and cytokine composition. Pro-tumorigenic chemo/cytokines such as IL-6, IL-10, CCL2, and CCL5 were decreased in NLRC5+ tumor-bearing ascites, correlating with significantly decreased frequency of tolerogenic DC2s subsets in this compartment. IL-12 and CXCL10 were also decreased in the ascites, which could partially explain why NLRC5 overexpression failed to confer antitumoral protection as a consequence of the lack of a proper pro-inflammatory milieu. However, it is not clear with the current data which cell types may be the source of these cytokines in this environment in this tumor model.

Previous reports have shown that in melanoma, MHC I and NLRC5 expressions correlate with a positive response to ICIs (47, 62). Although mice with NLRC5-overexpressing OC tumors did not have prolonged median survival when treated with an anti-PD-L1 antibody, the overall survival rate was increased in mice bearing NLRC5+ tumors, perhaps indicating some synergy achieved by TAA presentation and PD-L1 blockade. Moreover, the increased frequencies of CTLs in the TME generated by NLRC5, the increased endogenous TAA production, and the increased immunogenicity of OC cells overexpressing NLRC5 could all play a cooperative antitumoral role to increase responsiveness to immunotherapies.

Failure to achieve antitumoral protection in the peritoneal cavity may be related to the ID8 model itself and the fact that low mutational burden in OC limit T-cell antitumoral production (52). Some discrepancies have been reported in the antitumoral role of NLRC5. In non-small-cell lung cancer, NLRC5 seems to be a negative indicator of prognosis (63), whereas in other types such as endometrial cancer and melanoma, NLRC5 downregulation has been correlated with poor prognosis (20, 64, 65). Recently, Szymczak et al. (66) showed that NLRC5 modulates IFN-α responses of human pancreatic β cells, potentially increasing the production of self-peptides and chemokines that could amplify autoimmunity in type 1 diabetes. Santharam et al. (67) similarly found that EL-4 lymphoma cells overexpressing NLRC5 increase the production and repertoire of MHC-I-associated peptides. These observations align with our findings showing an increased antitumoral response to an infected cell vaccine overexpressing NLRC5.

Personalized cancer vaccines may be the future to eradicate cancer as some studies have demonstrated in OC (68). Whole tumor lysates overexpressing NLRC5 could achieve a better antitumoral response and T-cell clonality toward self-peptides that can be exploited in a dendritic cell vaccine delivery. NLRC5 can restore tumor immunogenicity by increasing MHC I allotypes, creating a potential avenue for therapeutic strategies to restore MHC I antigen presentation as a combinatorial approach with other therapies against primary tumors but also in the context of MHC I downregulation as an intrinsic mechanism for acquired resistance to immunotherapy in cancer patients (69).

## Data availability statement

The original contributions presented in the study are included in the article/**Supplementary Material**, further inquiries can be directed to the corresponding author.

## Ethics statement

The studies involving humans were approved by Ottawa Ovarian Cancer Tissue Bank, OHSN-REB Protocol #1999540-01H. The studies were conducted in accordance with the local legislation and institutional requirements. Written informed consent for participation in this study was provided by the participants. The animal study was approved by the Animal Care Committee at the University of Ottawa. The study was conducted in accordance with the local legislation and institutional requirements.

## Author contributions

GR: Conceptualization, Data curation, Formal analysis, Funding acquisition, Investigation, Methodology, Project administration, Resources, Software, Supervision, Validation, Visualization, Writing – original draft, Writing – review & editing. EY: Data curation, Formal analysis, Methodology, Software, Visualization, Writing – review & editing. HM: Methodology, Software, Writing – review & editing. VM: Data curation, Formal analysis, Methodology, Validation, Writing – review & editing. KJCG: Data curation, Methodology, Software, Writing – review & editing. AC: Data curation, Formal analysis, Methodology, Writing – review & editing. AH: Data curation, Methodology, Writing – review & editing. EM: Methodology, Resources, Writing – review & editing. SR: Methodology, Writing – review & editing. KG: Conceptualization, Data curation, Formal analysis, Methodology, Resources, Software, Validation, Writing – review & editing. BV: Data curation, Funding acquisition, Resources, Supervision, Writing – review & editing.
