# Supplementary material for: NLRC5 overexpression in ovarian tumors remodels the tumor microenvironment and increases T-cell reactivity toward autologous tumor-associated antigens
Source: Front Immunol. 2024 Jan 3;14:1295208. doi: 10.3389/fimmu.2023.1295208 (PMC10791902; doi:10.3389/fimmu.2023.1295208)
Supplement: Supplementary file 1 [file DataSheet_1.pdf]

SUPPLEMENTARY FIGURE 1

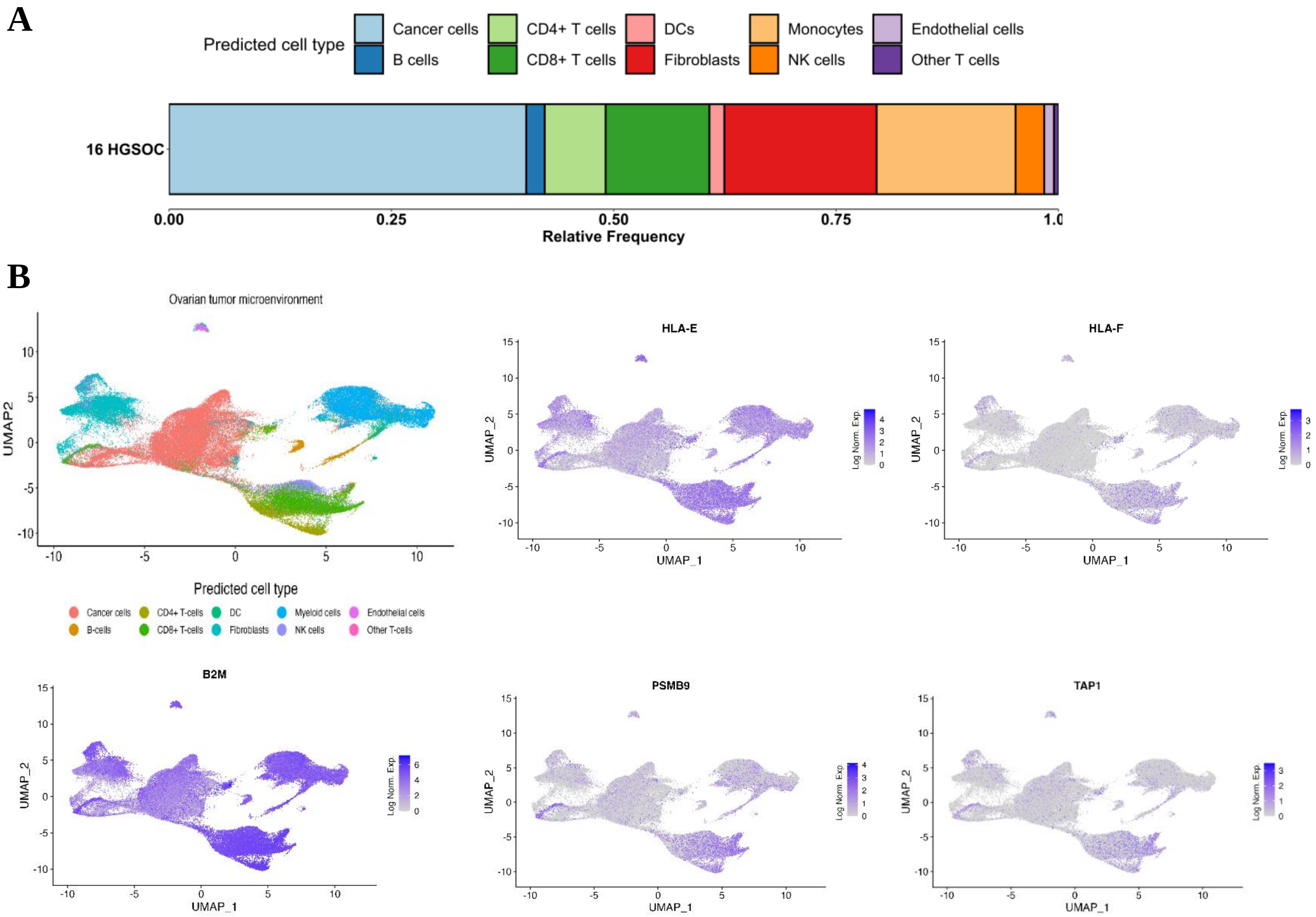

Fig. S1. Most of the antigen processing and presentation genes are downregulated in OC cells. Analysis of single-cell RNA-sequencing data from 16 ovarian tumors[24]. **(A)** Bar plot showing the overall cell proportions found in each cluster derived from the sc-RNAseq dataset. **(B)** UMAP analysis depicting cell clusters found in the TME of OC and overall expression of *HLA-E*, *HLA-F*, *B2M*, *PSMB9* and *TAP1*. Heatmap displays the level of expression in cell types.

SUPPLEMENTARY FIGURE 2

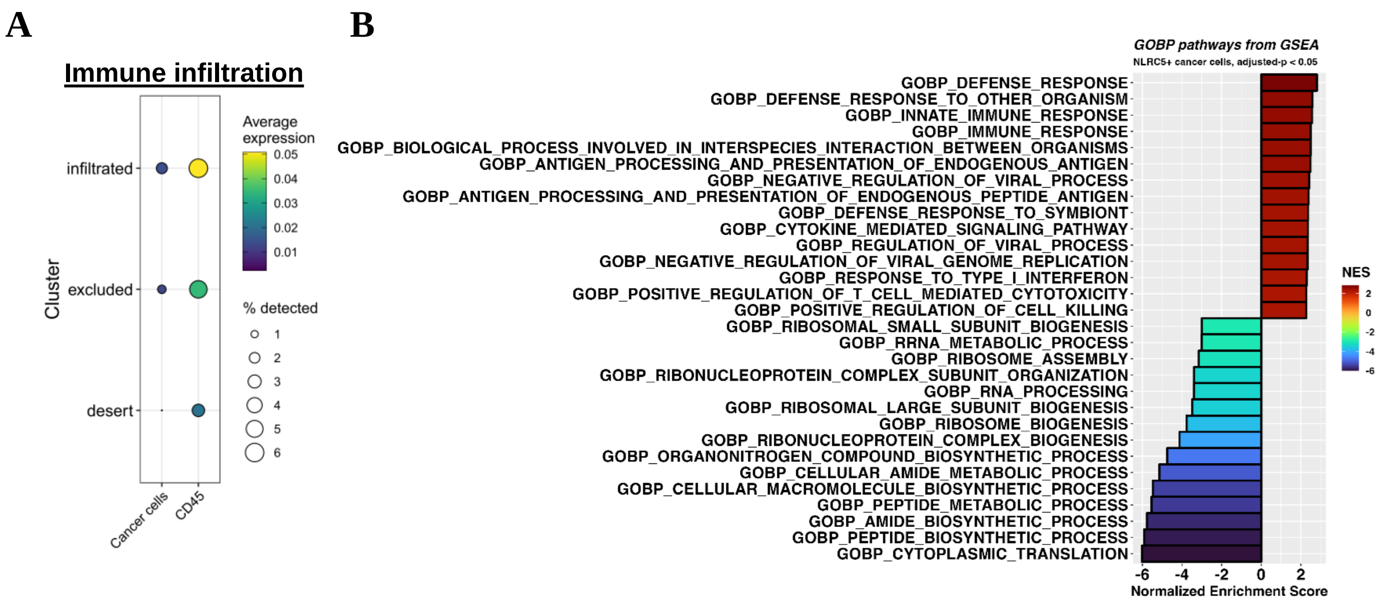

Fig. S2. NLRC5 expression correlates with an immune-infiltrated TME. Data analysis performed on cancer cells from the single-cell RNA-sequencing database of 16 ovarian tumors by Hornburg et al.[24]. **(A)** Relative frequency and level of expression of NLRC5 in cancer cells vs. CD45+ cells found in the OC TME analyzed by scRNA-seq[24]. Data are categorized by degree of immune infiltration. **(B)** GSEA of enriched biological pathways in high- and low-expressing *NLRC5+* cancer cells found in human ovarian tumors. GSEA analysis of biological pathways (GOBP) enriched by differentially expressed genes in *NLRC5+* cancer cells vs. *NLRC5-* cancer cells. Normalized enrichment scores (NES) represent either enriched or un-enriched pathways.

SUPPLEMENTARY FIGURE 3

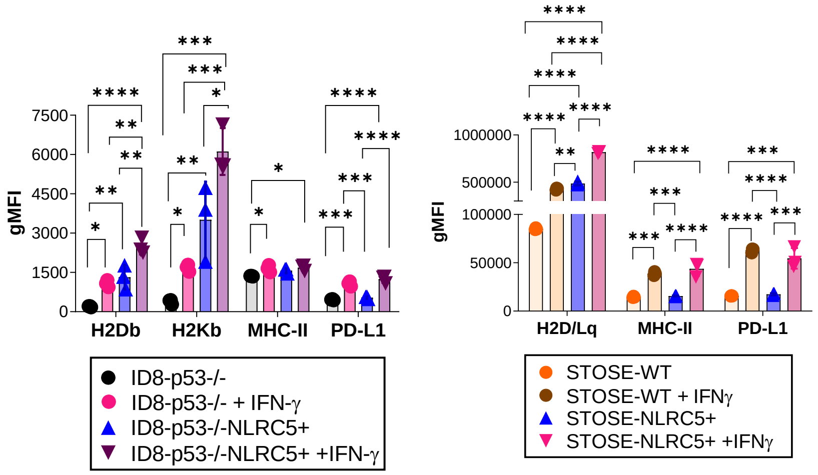

Fig. S3. NLRC5 overexpression in OC cells significantly increases MHC I expression. Quantification of the geometric mean fluorescence intensity (MFI) of H2Db, H2Kb (H2D/Lq for STOSE), MHC-II and PD-L1 protein expression assessed by flow cytometry on ID8-p53-/- or STOSE cell lines overexpressing or not NLRC5 under basal conditions or in presence of IFN-γ for 72 hours. Bar plots are representative of three independent experiments with three technical replicates. Cells were gated as singlet, viable cells. Significance was determined by one-way ANOVA with Tukey's multiple comparison test, *p<0.05, **p<0.01, ***p<0.001, ****p<0.0001.

SUPPLEMENTARY FIGURE 4

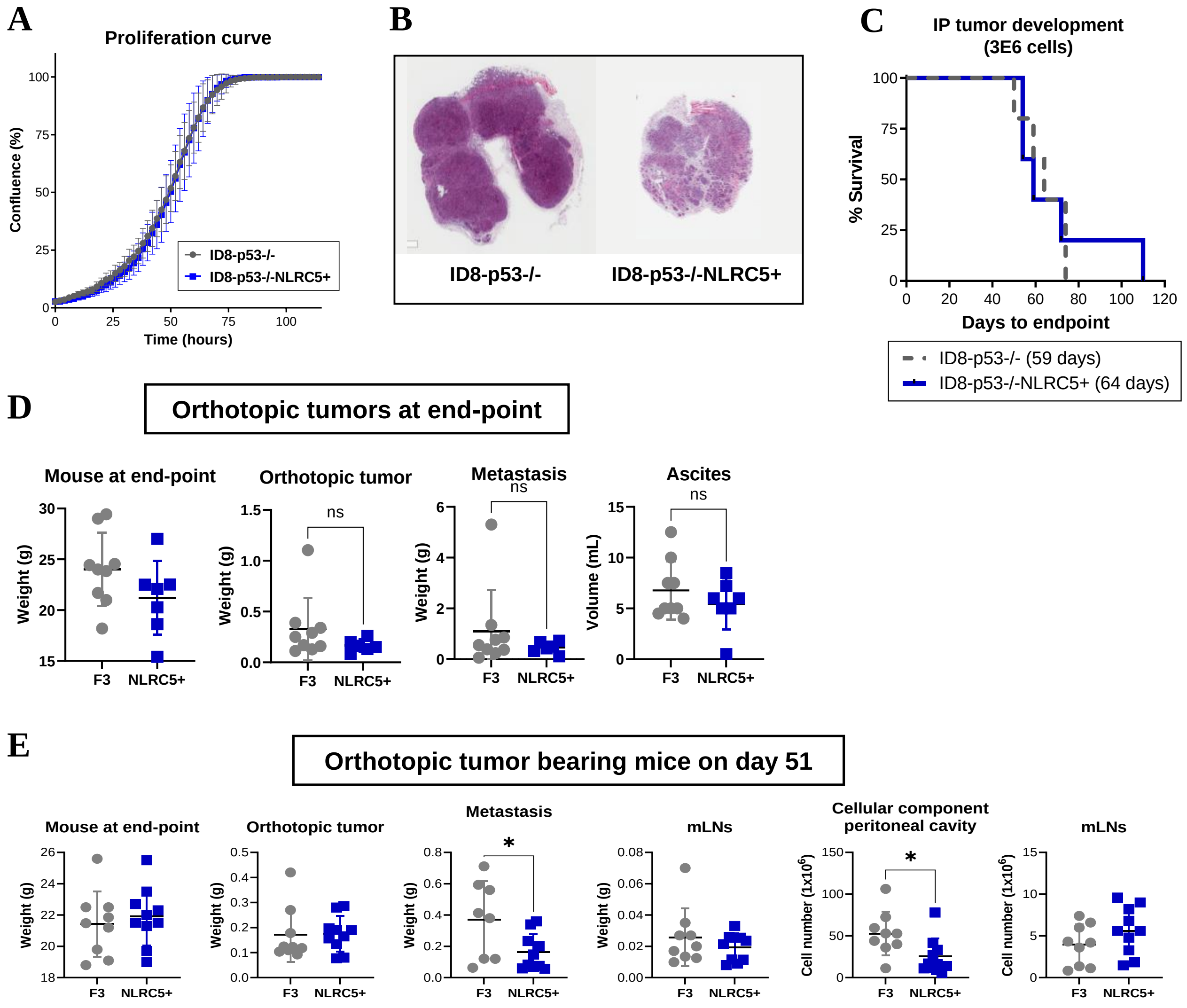

Fig. S4. Characterization of ID8-p53-/-NLRC5+ cell lines *in vitro* and *in vivo*. (A) *In vitro* proliferation of ID8-p53-/- and ID8-p53-/-NLRC5+ cells over time assessed using Incucyte. N=5 technical replicates per cell line. Error bars represent SD. **(B-E)** Necropsy characteristics for tissues collected from ID8-p53-/- (F3) or ID8-p53-/-NLRC5+ tumor-bearing mice injected with (B) $5x10^6$ cells injected subcutaneously (SC), (C) $3x10^6$ cells injected IP, and (D-E) 1.5 $x10^5$ cells under each ovarian bursa. (B) H&E staining of SC tumors representative of each cell line at 13 weeks after cell injection. (C) Survival Kaplan-Meier plots of ID8-p53-/- or ID8-p53-/-NLRC5+ tumor-bearing mice. Median survival is noted in parentheses. (D) Weights of mice, ovarian tumors and metastases, and volume of ascites collected at endpoint. (E) Weights of mice, ovarian tumors, metastases, and mesenteric LNs, and cell count of the peritoneal washes and the mesenteric LNs collected all on day 51 post-tumor cell injection. For orthotopic tumors n=10/cell type; for IP and SC tumors n=5/cell type. Data is representative of two independent experiments with similar results. Significance was determined by unpaired t-test, ns=not significant, *p<0.05.

SUPPLEMENTARY FIGURE 5

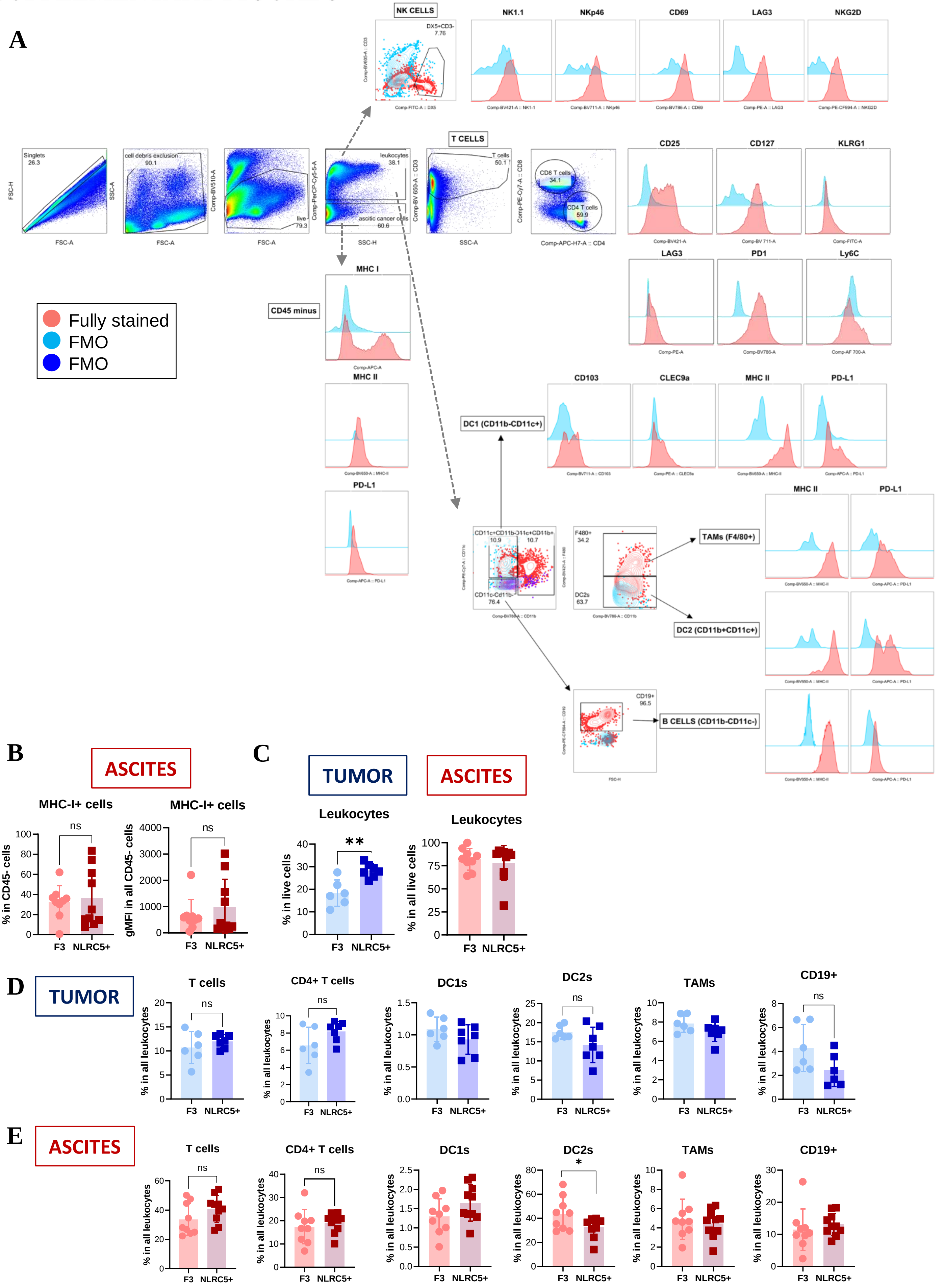

Fig. S5. Immune phenotyping of orthotopic OC tumors and ascites overexpressing NLRC5. Flow cytometric analysis of the immune composition of orthotopic tumors and ascites from mice bearing ID8-p53-/- (F3) or ID8-p53-/-NLRC5+ (NLRC5+) tumors. **(A)** Gating strategy employed to discriminate each immune cell subset: Singlets, Debris exclusion, Live Cells, CD45+ (Leukocytes), CD45- (non-immune cells/cancer cells). For CD45- population, the markers H2D/Kb (MHC I), MHC II, and PD-L1 were analyzed. For NK cells (DX5+CD3-) NK1.1, NKp46, CD69, LAG3, and NKG2D markers were assessed. For T cells (CD3+), CD8, CD4 markers were used and then CD25, CD127, Ly6C, PD1, KLRG1 and LAG3 were analyzed for each T cell subset. Antigen presenting cells (CD3-), were categorized as follows: CD11b+CD11c-F4/80+ (Tumor-associated macrophages, TAMs), CD11b-CD11c+ (DC1s), CD11b+CD11c+F4/80- (DC2s), CD11b-CD11c-CD19+ (B cells). CD103, CLEC9a, MHC II, and PD-L1 markers were assessed for all APCs. Dot plots or histograms depict protein expression for each marker. Red (fully stained), blue (Fluorescence minus one, FMOs) controls for each marker. **(B)** Percentage and geometric mean fluorescence intensity (gMFI) showing MHC I expression on CD45- cells found in the ascites. **(C)** Frequency of total leukocytes among live cells analyzed for each tumor (blue) and ascites (red) sample. **(D)** Frequency of T cells, CD4+ T cells, DC1s, DC2s, TAMs and B cells among all leukocytes in the tumors and **(E)** in the ascites. Data presenting n=6-7 orthotopic tumors/cell type, n=9-10 ascites/cell type, and n=9 mLNs/cell type. Significance was determined by unpaired t-test, ns=not significant, *$p<0.05$; **$p<0.01$.

SUPPLEMENTARY FIGURE 6

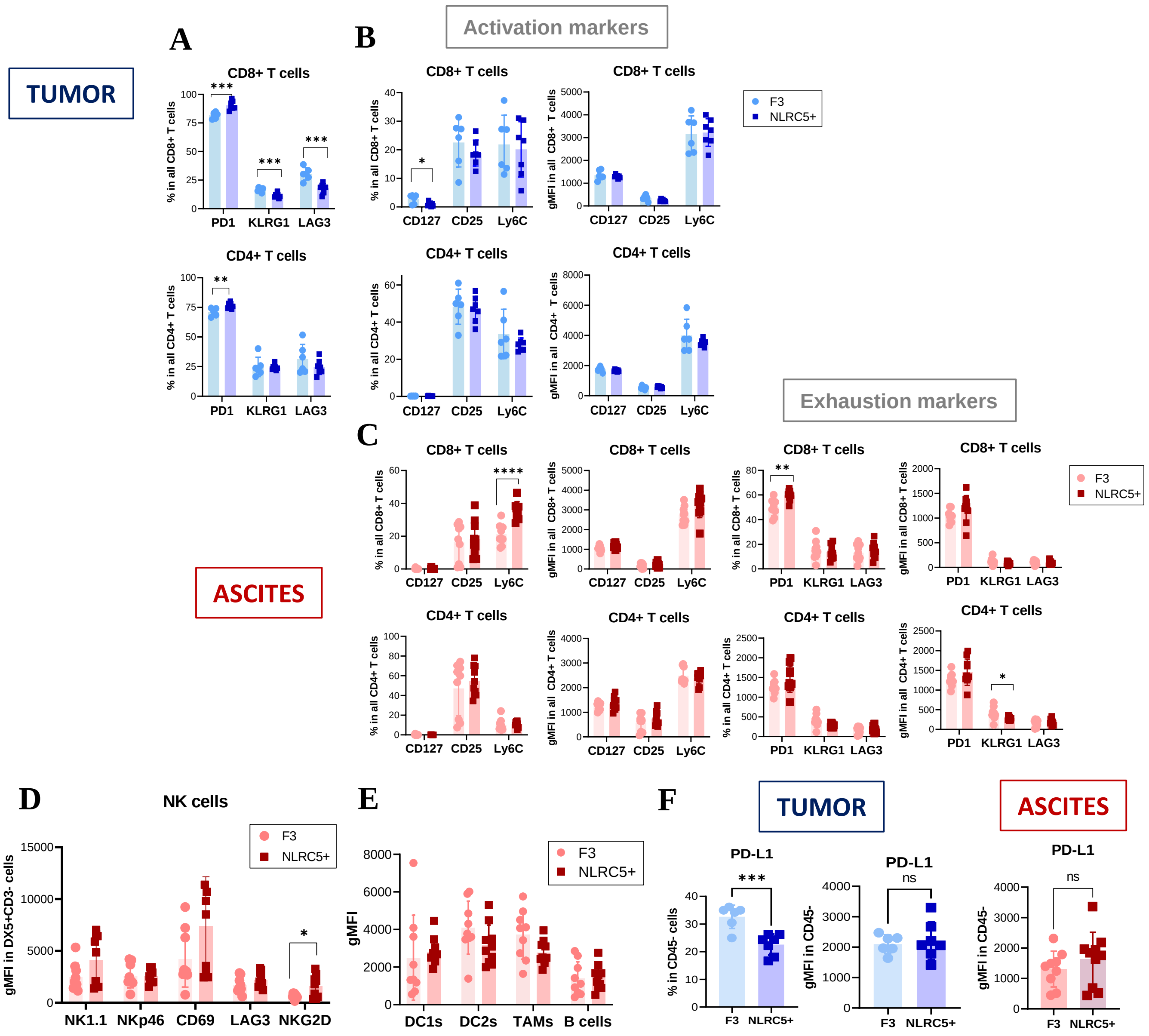

Fig. S6. Functional phenotype of immune subsets in OC orthotopic tumors and ascites overexpressing NLRC5. Flow cytometric analysis of orthotopic tumors and ascites from mice bearing ID8-p53-/- (F3) or ID8-p53-/-NLRC5+ (NLRC5+) tumors (as summarized in Fig. S5A). **(A)** Frequencies of PD1, KLRG1 and LAG3 on CD8+ (top panel) and CD4+ T cells (bottom panel) from the tumors. **(B)** Frequencies (left) and gMFI (right) of CD127, CD25 and Ly6C activation markers expressed on CD8+ (top panels) and CD4+ (bottom panels) T cells from the tumors. **(C)** Frequencies and gMFIs of CD127, CD25 and Ly6C activation markers, and PD-1, KLRG1 and LAG3 exhaustion markers expressed on CD4+ and CD8+ T cells from the ascites. **(D)** gMFI depicting the overall expression of NK1.1, NKp46, CD69, LAG3 and NKG2D in all DX5+CD3- cells from the ascites. **(E)** gMFI depicting the overall expression of PD-L1 in DC1s, DC2s, TAMs and B cells from ascites. **(F)** Frequencies and gMFI depicting the expression of PD-L1 in all CD45- cells from tumors (blue) and ascites (red). Data presenting n=6-7 orthotopic tumors/cell type, n=9-10 ascites/cell type. Significance was determined by unpaired t-test, ns=not significant, *p<0.05, **p<0.01, ***p<0.001, ****p<0.0001.

SUPPLEMENTARY FIGURE 7

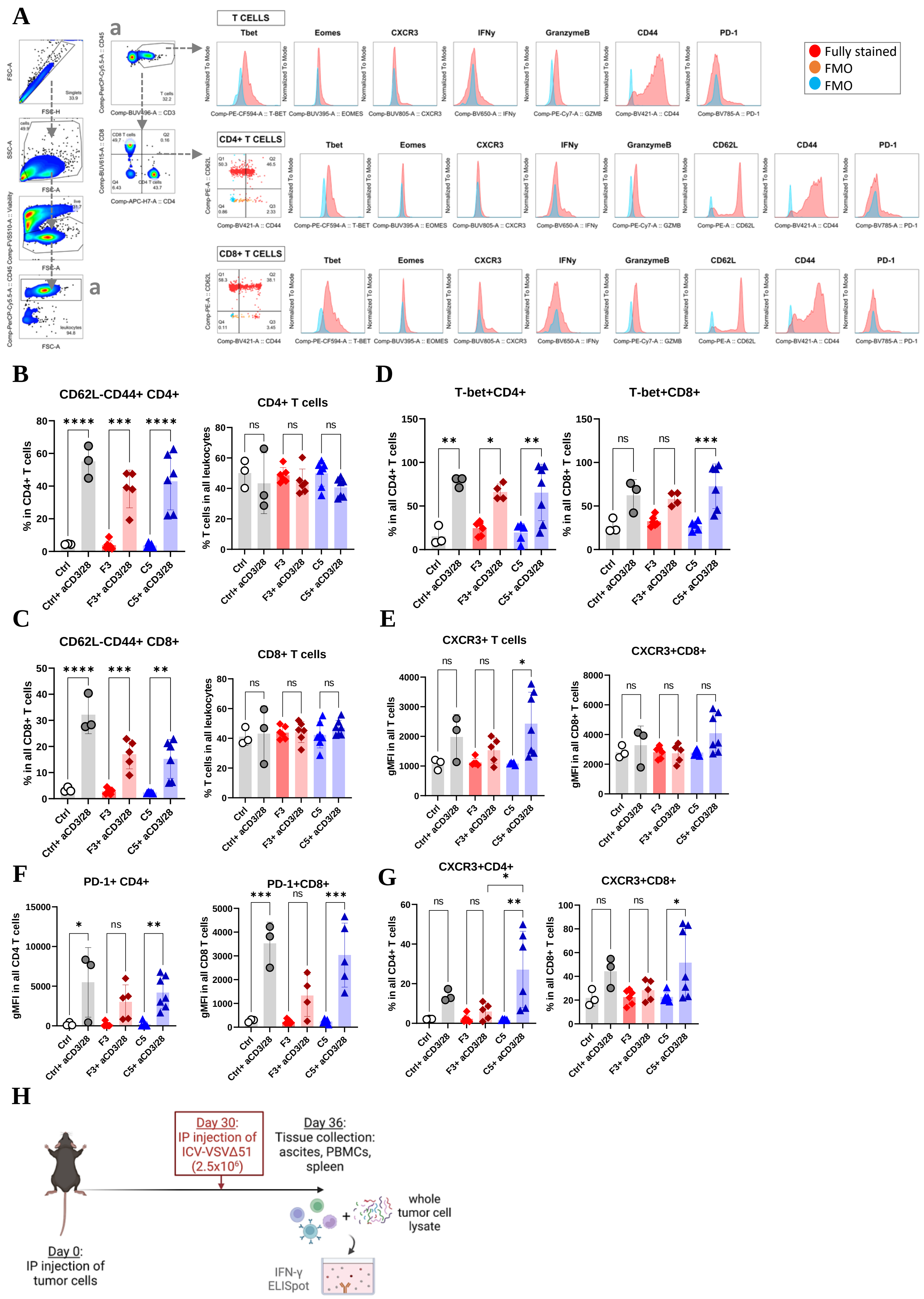

Fig. S7. Cytotoxic and effector phenotype of T lymphocytes from mice with tumors overexpressing NLRC5. C57BL/6 mice received either 5 $x10^6$ ID8-p53-/- (F3) or ID8-p53-/-NLRC5+ (NLRC5+) cells by IP injection. At day 50, mice were bled to analyze PBMCs by *ex vivo* activation by flow cytometry. **(A)** Gating strategy employed to discriminate the T cell compartment: Singlets, debris exclusion, live cells, CD45+ (leukocytes), T cells (CD3+), CD8, and CD4 markers were used and then T-bet, Eomes, CXCR3, IFN-γ, GranzymeB, CD44 and PD-1 were analyzed for each T cell subset. Dot plots or histograms depict protein expression for each marker. Red (fully stained), blue and orange curves represent (Fluorescence minus one, FMOs) controls for each marker. **(B-G)** T cells from PBMCs from tumor-bearing and tumor-naïve mice, activated *ex vivo* with antiCD3/CD28 and analyzed by flow cytometry. Bar graphs show the frequency or gMFI of protein expression for each marker in CD4+ and CD8+ T cells, and or total T cells (E). Each dot represents a biological replicate. n=6 red/dark red F3 (ID8-p53-/-) mice, n=7 blue/dark blue C5 (ID8-p53-/-NLRC5+), and n=3 white/gray tumor naïve mice were used as control. Significance was determined by one-way ANOVA, followed by Tukey's post-hoc test, ns=not significant, *$p<0.05$, **$p<0.01$, ***$p<0.001$, ****$p<0.0001$. **(H)** Experimental design for IFN-γ ELISpot (created with BioRender): tumor-naïve or tumor-bearing mice were challenged with an infected cell vaccine consisting of ID8-p53-/- or ID8-p53-/-NLRC5+ cells irradiated at 100Gy and infected *ex vivo* with VSVΔ51. Six days later, PBMCs were obtained from saphenous vein and assessed for IFN-γ ELISpot in the presence of whole cell lysates: Ag irrF3= from irradiated ID8-p53-/-; Ag irrC5= from irradiated ID8-p53-/-NLRC5+, VSV-N peptide was used as a specific control for VSV infection.

SUPPLEMENTARY FIGURE 8

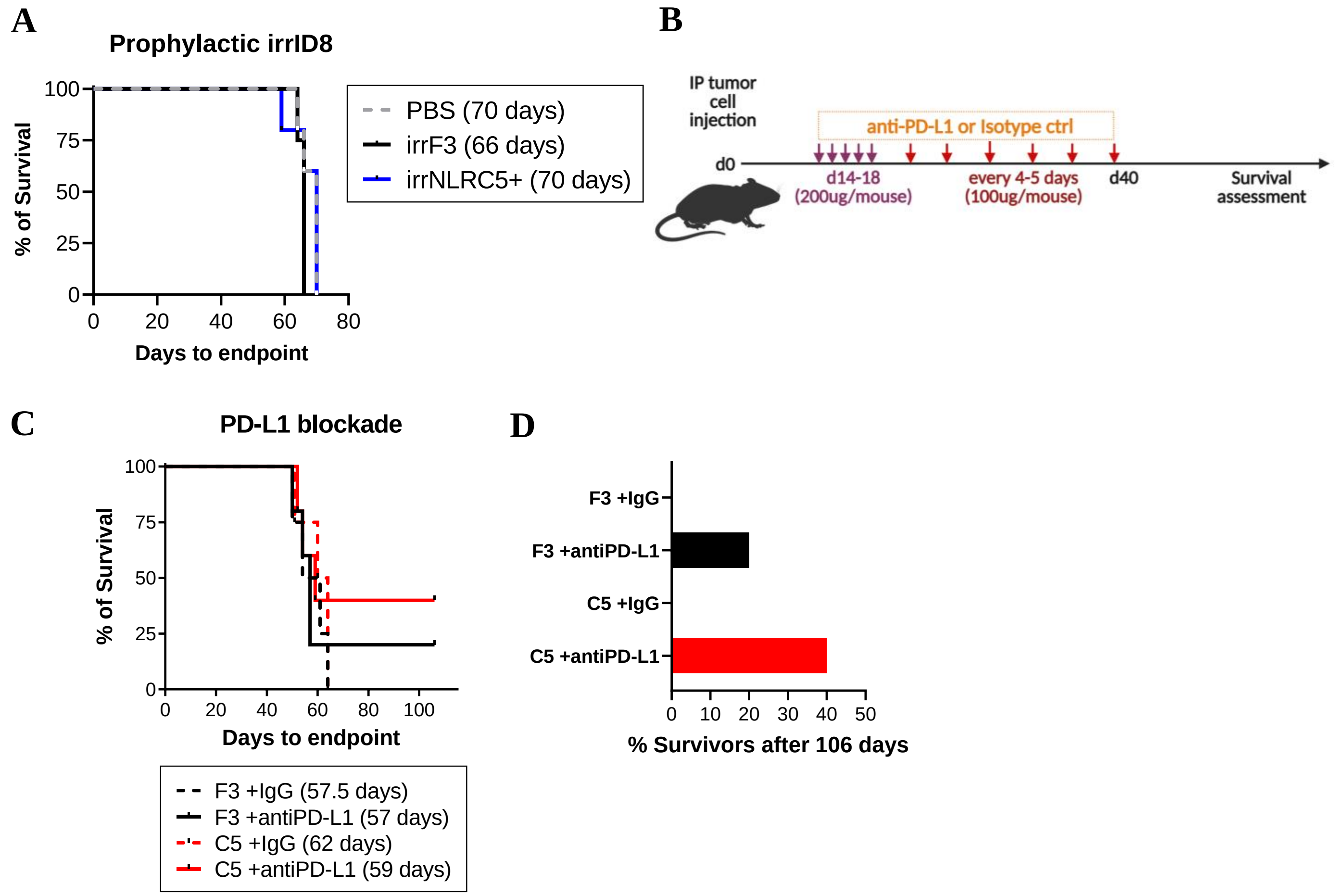

Fig. S8. NLRC5-overexpressing tumors have a greater response to PD-L1 blockade. (A) Survival of mice bearing ID8-p53-/- tumors and treated by IP injection on day 14 with irradiated ID8-p53-/- (irrF3) or ID8-p53-/-NLRC5+ (irrNLRC5+) cells, or PBS as control. Survival Kaplan-Meier plots depicting N=5 mice/treatment. Data are representative of two independent experiments with similar results. Median survival depicted in parenthesis. **(B-D)** Mice were injected IP with ID8-p53-/- or ID8-p53-/-NLRC5+ cells and treated with anti-PD-L1 or isotype control according to the schedule shown in (B). (C) Survival Kaplan-Meier plots and (D) percentage of survivors at day 106. N=10/cell type/treatment. Data representative of two independent experiments with similar results. Median survival depicted in parentheses.

SUPPLEMENTARY TABLE 1

Table S1. Human patient-derived OC cell line characteristics

Cell line	Origin	Known relevant mutations
**A2780cp** (RRID:CVCL_0135)	Ovarian Endometrioid adenocarcinoma - Selected for resistance to cisplatin[1]	ATM: P604S; PTEN: K128_R130del
**A2780s** (RRID:CVCL_4863)	Ovarian Endometrioid adenocarcinoma[1]	ATM: P604S; PTEN: K128_R130del
**OV-1946** (RRID:CVCL_4375)	Ovarian Serous (high grade) adenocarcinoma, derived from metastatic site (ascites)[2]	TP53: R273C
**OV-90** (RRID:CVCL_3768)	Ovarian Adenocarcinoma , derived from metastatic site (ascites)[3]	Gene fusion CDKN2D-WDFY2; SMAD4: R445Ter; TP53: S215R
**OVCA-420** (RRID:CVCL_3935)	Ovarian Serous adenocarcinoma (ascites)[4]	TP53: R273H
**OVCAR-8** (RRID:CVCL_1629)	Ovarian Serous (high grade) adenocarcinoma[5]	TP53: Y126-K132-/-; CTNNB1: Q26R; ERBB2: G776V; KRAS: P121H
**PEO1** (RRID:CVCL_2686)	Ovarian cystadenocarcinoma derived from metastatic site (ascites)[6]	TP53: G244D; BRCA2: Y1655Ter
**PEO4** (RRID:CVCL_2690)	Ovarian cystadenocarcinoma derived from metastatic site. Same individual as PEO1 cell lines[6]	TP53: G244D; BRCA2: Y1655Y; NF1: R160W
**SK-OV-3** (RRID:CVCL_0532)	Ovarian Serous cystadenocarcinoma, derived from metastatic site (ascites)[7–9]	TP53: H179R
**TOV-3041G** (RRID:CVCL_9T24)	Ovarian Serous (high grade) adenocarcinoma[10]	BRCA1/p53 silenced

References

1. Behrens, B. C. *et al.* Characterization of a cis-diamminedichloroplatinum(II)-resistant human ovarian cancer cell line and its use in evaluation of platinum analogues. *Cancer Res.* **47**, 414–418 (1987).
2. Ouellet, V. *et al.* Characterization of three new serous epithelial ovarian cancer cell lines. *BMC Cancer* **8**, 152 (2008).
3. Provencher, D. M. *et al.* Characterization of four novel epithelial ovarian cancer cell lines. *In Vitro Cell. Dev. Biol. Anim.* **36**, 357–361 (2000).
4. OVCA 420, human cell line STR and SNP profiles fro... - BioSample - NCBI. https://www-ncbi-nlm-nih-gov.proxy.bib.uottawa.ca/biosample/3471874.
5. Schilder, R. J. *et al.* Metallothionein gene expression and resistance to cisplatin in human ovarian cancer. *Int. J. Cancer* **45**, 416–422 (1990).
6. Langdon, S. P. *et al.* Characterization and properties of nine human ovarian adenocarcinoma cell lines. *Cancer Res.* **48**, 6166–6172 (1988).
7. Fogh, J., Wright, W. C. & Loveless, J. D. Absence of HeLa cell contamination in 169 cell lines derived from human tumors. *J. Natl. Cancer Inst.* **58**, 209–214 (1977).
8. Fogh, J., Fogh, J. M. & Orfeo, T. One hundred and twenty-seven cultured human tumor cell lines producing tumors in nude mice. *J. Natl. Cancer Inst.* **59**, 221–226 (1977).
9. Yaginuma, Y. & Westphal, H. Abnormal structure and expression of the p53 gene in human ovarian carcinoma cell lines. *Cancer Res.* **52**, 4196–4199 (1992).
10. Fleury, H. *et al.* Novel high-grade serous epithelial ovarian cancer cell lines that reflect the molecular diversity of both the sporadic and hereditary disease. *Genes Cancer* **6**, 378–398 (2015).

SUPPLEMENTARY TABLE 2

Table S2. Diagnostic and pathologic characteristics of OC patient's samples

Sample ID	Collection Date#	Histo-pathology	Grade	Chemo Line*	Chemo Protocol*	Chemo (Previous)	PFI (months)	Platinum resistance	Survival (months)
1344	12/5/2005	Serous	2	Post 3rd line	NA	Carbo/Taxol x 2<br>Cisplatin/Taxol	10	N	40
1359	9/4/2004	Serous	NA	Pre Sx/Pre Neo Chemo	NA	NA	NA	NA	35
1377	5/26/2004	Serous	Hi	3rd line	Taxol (Palliative - weekly)	Carbo/Taxol<br>Yondelis/Doxorubicin	2	Y	19
1454	9/1/2005	Serous	3	Post 1st line	NA	Carbo/Taxol	0	Y	7
1557	28/09/2009	Serous	2	Post 4th line	NA	Carbo/Taxol x 2<br>Lipodox; Topotecan	18	N	81
1846	5/30/2002	Serous	Hi	Post 3x 1st line/Pre Sx	Lipodox	NA	0	Y	4
1891	8/25/2004	Serous	NA	Pre Sx/Pre Neo Chemo	NA	NA	NA	NA	35
1908	10/18/2005	Serous	NA	Pre Sx/Pre Chemo	NA	NA	NA	NA	NA
1930	7/11/2007	Serous	NA	Pre Sx/Pre Neo Chemo	NA	NA	0	Y	13
2211	8/24/2017	MMMT	3	Pre Sx/ Neo Chemo	Carbo/Taxol	NA	NA	Y	6
2219	9/25/2017	Serous	3	3rd line	Carboplatin	Carbo/Taxol<br>Taxol/Cisplatin	5	Y	10
2272	11/16/2018	Serous	NA	Post 2nd line	NA	Carbo/Taxol<br>Caelyx switched to Gemcitabine	NA	NA	10

* At ascites's collection time
NA: information not available

#all patients were assessed as "progressing"

SUPPLEMENTARY TABLE 3

Spearman correlation coefficients and *p* values	
	NLRC5
CXCL10	0.6849 (p<1E-04)
CXCL11	0.6837 (p<1E-04)
IRF9	0.6797 (p<1E-04)
CCL5	0.6493 (p<1E-04)
GZMB	0.6332 (p<1E-04)
KLRK1	0.6289 (p<1E-04)
GZMA	0.6286 (p<1E-04)
CD8A	0.6151 (p<1E-04)
CD274	0.5901 (p<1E-04)
CD86	0.5541 (p<1E-04)
CXCL9	0.5187 (p<1E-04)
IRF7	0.5129 (p<1E-04)
OAS1	0.4875 (p<1E-04)
CXCR3	0.4799 (p<1E-04)
EOMES	0.4468 (p<1E-04)
CD4	0.4448 (p<1E-04)
PRDM1	0.3803 (p<1E-04)
TBX21	0.3736 (p=1E-04)
HLA-C	0.3384 (p=3E-04)
CD20	0.3328 (p=4e-04)
CCL2	0.3122 (p=9e-04)
IFNG	0.3044 (p=0.0012)
HLA-B	0.2669 (p=0.0046)
TNF	0.1876 (p=0.0487)
CD19	0.1776 (p=0.0622)
IL2RA	0.1519 (p=0.1114)
IFNB1	0.149 (p=0.1187)
IL10	0.1476 (p=0.122)
IRF3	0.1322 (p=0.1667)
IL12B	0.1259 (p=0.188)
HLA-A	0.1247 (p=0.1921)
IL2	0.0957 (p=0.3178)
FOXP3	0.0796 (p=0.4064)
NCAM1	-0.0596 (p=0.5344)
IFNA2	0.0166 (p=0.8625)
PRF-1	-0.0045 (p=0.9628)

Table S3. Spearman correlation of antitumoral immune related genes and *NLRC5* expression in OC tumors. Spearman correlation coefficient analysis of antitumoral immune-related genes and *NLRC5* was performed using the kmplot.com ovarian cancer database[28]. Affymetrix IDs: HLA-A (215313_x_at), HLA-B (209140_x_at), HLA-C (216526_x_at), CD8A (205758_at), NCAM1 (CD56, 212843_at), PRF-1 (218149_s_at), GZMB (210164_at), PDCD1 (PD-1, 207634_at), CD274 (PD-L1, 227458_at), IFNG (210354_at), TBX21 (T-BET, 220684_at), IL10 (207433_at), IL2 (207849_at), IFNB1 (208173_at), IL12B (207901_at), IL2RA (206341_at), PRDM1 (BLIMP-1, 228964_at), FOXP3 (224211_at), TNF (207113_s_at), KLRK1 (NKG2D, 205821_at), ITGB2 (LTA, 202803_s_at) and NLRC5 (226474_at). Spearman correlation coefficient analysis was performed on n=111 samples, serous subtype and P53 mutated status. *$p \leq 0.05$, **$p \leq 0.01$, ***$p \leq 0.001$, ****$p \leq 0.0001$.

SUPPLEMENTARY TABLE 4

Table S4. Average gene expression with two fold change in presence or absence of *NLRC5* expression in OC cells (excel file)

SUPPLEMENTARY TABLE 5

Table S5. qPCR primers used to analyze the expression of gene of interests

Gene ID	Gene name	Primer sequences	
Human Transcripts		**Forward**	**Reverse**
NM_032206.4	*NLRC5*	CTGGCTGGTGAACCCATCAT	AGACCCAGAATGGCTGAACG
NM_002116.8	*HLA-A*	TCCCTCACAGCTTGTAAAGTG	ATGCCTACACGAACACAGAC
NM_005514.8	*HLA-B*	GATGTAATCCTTGCCGTCGT	GCGGCTACTACAACCAGAGC
NM_002117	*HLA-C*	GGAGACACAGAAGTACAAGCGC	ACATCCTCTGGAGGGTGTGAGA
NM_004048.4	*B2M*	TCAATGTCGGATGGATGAAA	GTGCTCGCGCTACTCTCTCT
NM_014143.4	*PD-L1*	AAATGGAACCTGGCGAAAGC	GATGAGCCCCTCAGGCATTT
NM_021130.5	*PPIA*	TGCTGGACCCAACACAAATGGTTC	TGGTGATCTTCTTGCTGGTCTTGC
Mouse Transcripts		**Forward**	**Reverse**
NM_013683.2	*Tap1*	TTGGCTTACGTGGCTGAAGT	TGAGACAAGGTTGCCCGCTG
NM_011530.3	*Tap2*	TTCACCCTGCATCCTGGAAC	CAGAACCCGAGAACAGACA
NM_009735.3	*B2m*	ACGTAACACAGTTCCACCCG	CAGTCTCAGTGGGGGTGAAT
NM_010380.3	*H2db*	GGGAAACACAGAAAGCCAAG	AAGTCACAGCCAGACATCTG
MF352193.1	*H2dq*	GATCACGCAGATCGCCAAGGACAAT	CCGTCGTATGCGTACTGCTCGTACCC
NM_001001892.2	*H2k*	AGACACAGAAAGCCAAGGG	CACTTCACAGCCAGAGATCAC
NM_001033207.3	*Nlrc5*	TCAGCCCAGAACAAGTATCC	TGGGCACAGACTTCCATTAG
NM_013585.2	*Psmb9*	AGAAGTCCACACCGGGACAA	TCAGCTATGGCTTGGGCATC
NM_010724.2	*Psmb8*	GAAGAGGCCTACGACCTTGG	CTCCACTTTCACCCAACCGT
NM_001205313.1	*Stat1*	GCCGAGAACATACCAGAGAATC	GATGTATCCAGTTCGCTTAGGG
NP_031501.1	*Rplp0*	TGACATCGTCTTTAAACCCCG	TGTCTGCTCCCACAATGAAG
NM_008907.2	*Ppia*	AGGGTGGTGACTTTACACGC	GATGCCAGGACCTGTATGCT

SUPPLEMENTARY TABLE 6

Table S6. Flow cytometry antibodies

	Flow antibodies	Supplier
**Mouse antibodies**	Fc-blocking CD16/CD32 (clone 2.4G2)	BD Biosciences
Extracellular markers	CD45-PerCP-Cy5.5 (clone 30-F11),	BD Biosciences
	CD3-BV650 or –BV605 (clone 145-2C11),	BD Biosciences
	CD4-FITC or –APC-H7 (clone GK1.5),	BD Biosciences
	CD8-PE-Cy7 (clone 53-6.7),	BD Biosciences
	PD-1-BV785 (clone 29F.1A12)	BioLegend
	LAG-3-PE (clone C9B7W)	BD Biosciences
	CD127-BV711 (clone SB/199)	BD Biosciences
	KLRG1-AF488 (clone 2F1)	BD Biosciences
	Ly6C-AF700 (clone AL-21)	BD Biosciences
	CXCR3-BUV805 (clone CXCR3-173)	BD Biosciences
	CD62L-PE (clone MEL-14)	BD Biosciences
	CD44-BV421 (clone IM7)	BD Biosciences
	CD69-BV786 (clone H1.2F3)	BD Biosciences
	CD49b-FITC (clone DX5)	BD Biosciences
	NK1.1-BV421 (clone PK136)	BD Biosciences
	NKp46-BV711 (clone 29A1.4)	BD Biosciences
	NKG2D-PE-CF594 (clone CX5)	BD Biosciences
	MHC-II-BV650 (clone M5/114.15.2)	BD Biosciences
MHC-I (C57BL/6 background)	H2Kb-BV421 (clone AF6-88.5)	BD Biosciences
MHC-I (C57BL/6 background)	H2Db-BV786 (clone KH95)	BD Biosciences
MHC-I (C57BL/6 background)	H2D/Kb-APC (clone 28-8-6)	BioLegend
MHC-I (FVB/N background)	H2D/Lq-BV711 (clone KH117)	BD Biosciences
	Gr1-BV605 (clone RB6-8C5)	BD Biosciences
	CD11b-BV786 (clone M1/70)	BD Biosciences
	CD11c-PE-Cy7 (clone HL3)	BD Biosciences
	PD-L1-PE or APC (clone MIH5)	BD Biosciences
	CD103-BV711 (clone 2E7)	BioLegend
	Clec9a-PE (clone 7H11)	BD Biosciences
	F4/80-BV421 (clone T45-2342)	BD Biosciences
	CD19-PE-CF594 (clone 1D3)	BD Biosciences
Intracellular markers	Eomes-BUV395 (clone X4-83)	BD Biosciences
	T-bet-PE-CF594 (clone O4-46)	BD Biosciences
	IFN-γ-BV650 (clone XMG1.2)	BioLegend
	GranzymeB-PE-Cy7 (clone QA18A28)	BioLegend
**Human antibodies**	HLA-ABC-PE (clone EMR8-5)	BD Biosciences
	PD-L1-BV421 (clone MIH1)	BD Biosciences

SUPPLEMENTARY TABLE 7

Table S7: Absolute numbers of NLRC5 expressing cells in the OC TME derived from sc-RNAseq analysis Hornburg dataset[24].

	Immune phenotype	NLRC5_status	n	% frequency of expression
1	desert	expressing	305	0.995560778
2	desert	zero	30331	99.00443922
3	excluded	expressing	674	4.316638914
4	excluded	zero	14940	95.68336109
5	infiltrated	expressing	1171	7.268325988
6	infiltrated	zero	18358	94.00378924
